# Uncovering the Inhibitory Molecular Mechanism of Pomegranate Peel to Urinary Bladder Urothelial Carcinoma Using Proteomics Techniques

**DOI:** 10.3390/life12111839

**Published:** 2022-11-09

**Authors:** Kuan-Hua Huang, Ching-Ping Chang, Lan-Hsiang Chien, Chien-Feng Li, Ling-Yu Tang, Yu-Yi Chan, Ting-Feng Wu

**Affiliations:** 1 Division of Urology, Department of Surgery, Chi-Mei Medical Center, Tainan 710, Taiwan; 2 Department of Medical Sciences Industry, College of Health Sciences, Chang Jung Christian University, Tainan 711, Taiwan; 3 Department of Medical Research, Chi-Mei Medical Center, Tainan 710, Taiwan; 4 Department of Biotechnology and Food Technology, Southern Taiwan University of Science and Technology, Tainan 710, Taiwan

**Keywords:** pomegranate, proteomics, HSP90, PTP1B, bladder cancer, urinary bladder urothelial carcinoma, PEPE2

## Abstract

Pomegranate (*Punica granatum* L.) fruit demonstrates the repressive effectiveness of many tumors. Our previous studies showed that the PEP (pomegranate peel extract) E2 fraction obtained from the ethyl acetate layer of the pomegranate peel’s ethanol extract exhibited the highest inhibitory activities to induce Urinary bladder urothelial carcinoma (UBUC) cell apoptosis. The ethyl acetate layer could lower the volume and weight of T24 tumors and initiate apoptosis in nude mice xenografted bladder tumors. In this study, we intended to clarify the inhibitory molecular process of Taiwanese local pomegranate peel to urinary bladder urothelial carcinoma using a proteomics strategy. Gel-based proteomics (two-dimensional gel electrophoresis coupled with tandem mass spectrometry) was used to get an insight into the molecular mechanisms initiated by PEPE2 to evoke bladder cancer cell apoptosis. We found eleven down-regulated and eight up-regulated proteins in PEPE2-treated T24 cells. Our results implied that these PEPE2-dysregulated proteins belong to cell apoptosis, cell proliferation, death receptor signaling, JAK/STAT signaling, the PPAR pathway, the PPARα/RXR α pathway, Rho family GTPase signaling, and RhoGDI signaling. In addition, HSP90 and PTP1B proteins, associated with apoptosis, were de-regulated in xenografted bladder tumors in nude mice fed with an ethyl acetate layer of ethanol extract. The findings above implied that pomegranate might be a potential chemopreventive resource for UBUC carcinogenesis.

## 1. Introduction

Urinary bladder urothelial carcinoma (UBUC) is the utmost common urinary system tumor globally and is the ninth most common cancer diagnosed in male Taiwanese in 2017 [1,2]. In the United States, UBUC is the fourth most common and eighth most lethal tumor among men [1]. Urothelial carcinoma is the most prevailing tumor among various bladder cancer varieties, accounting for over 90% of bladder tumor incidence in developed countries [1]. Based on the WHO classification (2004), UBUC cells can be recognized as low or high grade. The majority of UBUCs demonstrate papillary and non-invasive or superficially invasive tumors that are often treated by curettage. Nevertheless, local recurrence often happens in some instances of UBUCs, even after fatal distal scattering [3]. It may be advantageous for cancer patients or high-risk persons to utilize a chemopreventive product for bladder cancer to prevent recurrence or prevalence regarding cancer management.

Pomegranate (*Punica granatum* L.) is a consumable fruit that grows in America, Northern India, Europe, and the Mediterranean countries. However, in Taiwan, it is primarily a horticultural plant. The pomegranate fruit is recognized to have ruby peels and white to deep red arils embedded in a membrane adherent to a spongy-like mesocarp enclosed by the rind. The pomegranate peel (membrane, mesocarp, and rind) is an abundant source of ellagitannins (mostly punicalagin), flavonoids, phenolics, and proanthocyanins [4]. The pomegranate arils, including seeds and juice, are edible and have robust anti-oxidant characteristics owing to anthocyanins and highly hydrolyzable tannins [4]. Pomegranate juice (PJ) contains a high level of flavonoids and phenolics, essentially anthocyanins [4].

Many established findings unveiling the cancer-preventive effects of pomegranate fruit have illustrated its mitigating efficacy to a variety of cancers. Pomegranate fruit extract (PFE) isolated from the eatable parts (pulp juice and seed coats) using 70% (*v*/*v*) acetone evokes lung tumor cell A549 apoptosis yet exhibits the most negligible detrimental influences on normal bronchial epithelial cells [5]. In addition, PFE therapy arrests A549 cells at G_0_/G_1_ through the down-expressions of cell cycle regulatory proteins operating in the G_1_ stage. Furthermore, PFE administration retards several signaling pathways, including the NF-κB pathway and various MAP kinase pathways, e.g., p38, phosphoinositide-3-kinase (PI3K)/protein kinase B (Akt), c-jun N-terminal kinase (JNK), and extracellular signal-regulated kinase (Erk) [5]. In A/J mice, PFE treatment dwindles the expansion of NTCU or B(a)P-initiated lung cancer. PFE-treated mice show decreased activities in MAP kinase cascades, mammalian target of rapamycin (mTOR) cascades, and NF-κB, inhibiting tumor cell proliferation and angiogenesis [6]. Besides lung cancer, PFE has been studied in cell culture and animal models as a potential chemotherapeutic candidate for human prostate cancer. Malik et al. (2005) [7] showed that PFE incubation provokes apoptosis in highly aggressive PC3 cells and under-regulates the cell cycle regulatory effector proteins—cyclins D1, D2, E cdk2, and ckd6—while over-regulating p21 and p27, leading to a dwindling in Bcl-2 concomitant with a surge in Bax. Malik et al. also demonstrated that incorporating PFE in the drinking water of nude mice xenografted with CWR22Rν1 cells gives tumor growth inhibition, which manifested in a decrease in the presence of prostate-specific antigen (PSA). Furthermore, orally feeding TRAMP mice 0.1 or 0.2% PFE in drinking water can appreciably impede prostate carcinogenesis by hindering the insulin-like growth factor-1 (IGF-1)/Akt/mTOR pathway [8]. Lee et al. (2012) indicated that Taiwanese local pomegranate juice provokes the apoptosis of PCa cells by the external death receptor and internal mitochondrial cascades. It can also dysregulate genes’ level of expression relating to NF-κB, apoptosis, metabolism, and cytoskeleton in PJ-given PCa cells [9]. Treatment of LAPC4 PCa cells with POMx, extracts made from the Wonderful variety of pomegranates, resulted in cell proliferation inhibition and apoptosis induction [10]. In addition, co-treatment with IGF-1 and POMx inhibits apoptosis in CWR22Rν1 cells induced by POMx. However, the impacts of IGF-1 in impeding POMx-initiated apoptosis are restored in the IGF-1 receptor null MEF cells, which thus implies the importance of IGF1 in antagonizing the therapeutic effects of POMx [10]. Retting et al. (2008) [11] indicated that using PE, a polyphenol/ellagitannin-abundant extract purified from pomegranate peels, could restrain androgen-independent LAPC4 xenografted tumor growth under the inhibition of the NF-κB signaling pathway. Clinical investigation in surgery or radiotherapy-cured prostate cancer (PCa) patients with increment prostate-specific antigen (PSA) showcased that drinking 8 ounces of pomegranate juice (PJ) (Wonderful variety, 570 mg total polyphenol gallic acid equivalents) daily meaningfully prolongs the PSA doubling time (PSADT) from 15 to 54 months [12]. However, a randomized, double-blind, and placebo-compared clinical examination demonstrated that the PFE cure does not delay the PSADT in PCa patients with increment PSA following the primary remedy compared to the placebo-controlled group [13]. The findings above imply that pomegranate may be a potential chemopreventive or chemotherapeutic resource for UBUC carcinogenesis and recurrence.

Our previous studies showed that the peel ethanol extract’s ethyl acetate (EtOAc) layer reveals the best repressing activity against bladder cells. One of the eight fractions (PEPE2 fraction) obtained from the EtOAc layer shows the highest inhibitory activity, and the PEPE2 fraction’s effectiveness is attributed to the UBUC cell apoptosis. Data on xenografted bladder tumors in nude mice show that feeding the EtOAc faction (2, 5, 10, and 100 mg/kg) could lower the volume and weight of T24 tumors and initiate apoptosis in the xenografted tumor [14]. In this study, gel-based proteomics (two-dimensional gel electrophoresis coupled with tandem mass spectrometry) was exploited to get insight into the molecular mechanisms initiated by PEPE2 to evoke bladder cancer cell apoptosis. Our results implied that PEPE2 might dysregulate the proteins involved in cell apoptosis, cell proliferation, death receptor signaling, JAK/STAT signaling, PPAR pathway, PPARα/RXR α pathway, Rho family GTPase signaling, and RhoGDI signaling to provoke cancer cell apoptosis.

## 2. Materials and Methods

### 2.1. Cell Lines

Dr. Chien-Feng Li gifted human UBUC J82 cells (high grade) from the Department of Pathology, Chi-Mei Medical Center, Tainan, Taiwan, and J82 cells were cultivated in Dulbecco’s Modified Eagle Medium with 10% (*v*/*v*) fetal bovine serum (FBS) (GIBCO, Grand Island, NY, USA) under 5% CO_2_ (*v*/*v*) at 37 °C in a CO_2_ incubator (CO-150, New Brunswick Scientific, Edison, NJ, USA). We procured the human UBUC T24 cells from the Bioresource Collection and Research Center, Hsinchu, Taiwan, and the T24 cells were cultured in McCoy’s5A (GIBCO, Grand Island, NY, USA) medium with 10% (*v*/*v*) FBS at 37 °C.

### 2.2. Preparation of Protein Lysates

Seventy to eighty percent of the confluent T24 cells were treated with 0.5% (*v*/*v*) DMSO containing PEPE2 with a final concentration of 20 μg/mL or 0.5% (*v*/*v*) DMSO for 24 h. Then, the treated cells were collected with 1000× *g* centrifugation for 5 min at 25 °C and destroyed by lysis buffer [2 M thiourea (JT Baker, Center Valley, PA, USA), 7 M urea (JT Baker, Center Valley, PA, USA), 4% (*v*/*v*) 3-[(3-cholamidopropyl) dimethylammonio]-1-propanesulfonate (CHAPS) (aMResco, Solon, OH, USA), 1 mM phenylmethanesulfonylfluoride (PMSF) (aMResco, Solon, OH, USA), 100 mM dithiothreitol (DTT) (USB Corcorporation, Cleveland, OH, USA), 40 mM Tris-base (pH 10) (aMResco, Solon, OH, USA), and one Complete Mini protease inhibitor cocktail tablet (Roche, Diagnostics, Indianapolis, USA) per one liter] under continuous mixing at room temperature for one hour. After lysis, a Type 90 Ti rotor (Beckman Coulter, Fullerton, CA, USA) was implemented to centrifuge the lysates at 349,000× *g* for 2 h at 15 °C to clear the nucleic acid and other interfering substances. According to the manufacturer’s procedure, the 2-D Clean-up Kit (GE Healthcare Bio-Sciences AB, Uppsala, Sweden) was used to precipitate the proteins in the supernatant. The MRB buffer [4% (*v*/*v*) CHAPS and 7 M Urea] was exploited to mingle the resultant pellet from the 2-D Clean-up Kit. Then, the solution was kept at room temperature for one hour while being rocked to dissolve the proteins. After incubation, the Bio-Rad DC protein assay quantitated the lysate protein concentration. Then, a proper quantity of DTT, IPG buffer, and thiourea were included in the lysate to convert the MRB buffer to the rehydration buffer [2 M thiourea, 7 M urea, 4% (*v*/*v*) CHAPS, 1 mM DTT, 0.5% (*v*/*v*) immobilized pH gradient (IPG) buffer (pH 4–7), and 0.1 (*w*/*v*) bromophenol blue (aMResco, Solon, OH, USA)]. Then, the lysates were kept at room temperature and rocked for one more hour. The lysed protein solutions were frozen at −80 °C for future experiments.

### 2.3. Two-Dimensional Gel Electrophoresis (2-DE)—Isoelectric Focusing (IEF) and SDS-Polyacrylamide Gel Electrophoresis (SDS-PAGE)

IEF and SDS-PAGE were performed as described by Shen et al. [15]. The BioRad Protean IEF Cell was first used to rehydrate the 18 cm immobiline^TM^ dry strips (pH 4–7) (GE Healthcare Bio-Sciences AB, Uppsala, Sweden) with a 300 µL rehydration buffer containing 200 µg of protein lysates made from mock and PEPE2-treated cells for 16 h at 20 °C. After rehydration, the proteins were concentrated at 20 °C under 50, 100, 200, 500, 1000, 5000, and 8000 V, respectively, with 81,434 voltage hours. Then, an equilibration buffer [2% (*w*/*v*) SDS (aMResco, Solon, OH, USA), cotaining 30% (*v*/*v*) glycerol (Kanto Chemical, Portland, OR, USA), and 6 M urea (aMResco, Solon, OH, USA)] with 2% (*w*/*v*) DTT (USB Corcorporation, Cleveland, OH, USA) was implemented to equilibrate the gel strips for 15 min. Afterwards, the DTT-equilibrated strips were incubated for 15 min with an equilibration buffer comprising 5% (*w*/*v*) iodoacetamide (aMResco, Solon, OH, USA). Then, the strips were put above a 12.5% (*w*/*v*) polyacrylamide gel sealed with 0.5% (*w*/*v*) agarose (aMResco, Solon, OH, USA) gel, and various proteins were resolved at 420 V using BioRad Protean IIxi until bromophenol blue run near the bottom.

### 2.4. Silver Staining

The 2D plus one silver staining kit (Amersham-Pharmacia Biotech, Amersham, UK) was used to detect proteins using a modified protocol described elsewhere [15]. Briefly, fixation solution (ethanol/water/acetic acid, 4/5/1, *v*/*v*) fixed the gel after electrophoresis, and the gels were treated with sensitizing solutions (0.5 M sodium acetate, 0.5% sodium thiosulphate) for 30 min. After sensitization, the gels were washed with ionized water for 10 min three times, incubated in a 0.25% silver nitrate solution for 20 min, and then developed through incubation with the developing solution (2.5% sodium carbonate and 0.015% (*v*/*v*) formaldehyde) till the protein spots appeared. Glutardialdehyde was not used in staining the gels used for MS.

### 2.5. Image Analysis and Statistical Analysis

PDQuest 8.0.1 (BioRad, Hercules, CA, USA) compared six pairs of well-resolved gel images obtained from untreated and PEPE2-treated T24 cells to find the dysregulated proteins. Abnormally expressed protein dots, as revealed by computer analysis, were further confirmed by eyes. Each spot’s intensity (volume) was measured and normalized as a percent (ppm) of all the powers of all protein spots in a gel (total normalized volume). When reaching normal distribution, the normal distribution investigation, and subsequently, Student’s *t*-Test (STATISTICA Version 10.0 MR1, StatSoft, Tulsa, OK, USA) analyzed the corrected intensities of individual dysregulated protein points of all the repetitive gels collected from the control and PEPE2-exposed T24 cells. However, log transformation was first used when normal distribution was improper, followed by the normal distribution test and then Student’s *t*-Test. The normalized volume of each spot in PEPE2-treated cells was assessed to that of the same spot in untreated cells. The confident statistical level within 95% (Student’s *t*-Test; *p* < 0.05) was recognized as meaningfully different in all analyses. Furthermore, the abnormally produced proteins observed in at least four of six gel pairs were galectin-1-related proteins.

### 2.6. Protein Recognition

As described previously, in-gel digestion and mass spectrometric protein identification were conducted [16]. Concisely, the peptides within a protein digest were determined using the LTQ-Orbitrap hybrid tandem mass spectrometer (ThermoFisher, Waltham, MA, USA) connected to the Agilent 1200 nanoflow HPLC system with an LC Packing C18 PepMap 100 column (length: 5 mm; internal diameter: 300 µm; bead size: 5 µm) as the trap column and Agilent ZORBAX XDB-C18 column (length: 50 mm; internal diameter: 75 µm; bead size: 3.5 µm) as the separation column. File Converter in the Xcalibur 2.0SR package (ThermoFisher, Waltham, MA, USA) and an in-house program acquired the MS/MS data, the charge, and the mass of each examined peptide. TurboSequest program (ver. 27, rev. 11; Thermo Fisher Scientific, Waltham, MA, USA) determined the best-accorded peptides from non-redundant FASTA protein sequences downloaded from the National Center for Biotechnology Information (ftp://ftp.ncifcrf.gov/pub/nonredun/ (accessed on 12 October 2010)) on 12 October 2010 with 541,927 entries. The peptides with ≤2 missed tryptic cuts were noted. When the database matched, the mass ranges covered 1 and 3.5 *m*/*z* for the fragment and precursor ions. When at least two peptides matched the theoretical proteins in the database, and matching data ensured a high Xcore (i.e., ≥2.0 for peptides with doubly charged and ≥3.0 for peptides with triply charged) and minimal disparities between the experimental and hypothetical masses (i.e., ∆M < 10 ppm), the protein identities were documented. For each MS/MS experiment, 25 fmol of bovine serum albumin (BSA) (aMResco, Solon, OH, USA) in the gel was used simultaneously to confirm the accuracy of the whole protein recognition protocol. The matching results were considered when the co-processed BSA samples obtained over 70% protein coverage and a ten ppm mass accuracy.

### 2.7. Western Immunoblotting

T24 or J82 cells were treated with 0.5% (*v*/*v*) DMSO containing PEPE2 with a final concentration indicated in figures or 0.5% (*v*/*v*) DMSO. The treated cells were collected and lysed in the lysis buffer [0.32 M sucrose (Avantor Performance Materials, Center Valley, PA, USA), 1% (*v*/*v*) Triton X-100 (Merck Millipore, Darmstadt, Germany), 5 mM EDTA (Merck Millipore, Darmstadt, Germany), 2 mM DTT (USB Corporation, Cleveland, OH, USA), 1 mM PMSF, 10 mM Tris (pH 8.0)]. Following quantitation of the protein amount using the Bio-Rad DC protein assay kit, an equivalent amount of 2× sample buffer (2% (*w*/*v*) SDS, 0.1 M Tris (pH 6.8), 10% (*v*/*v*) glycerol, 0.2% (*v*/*v*) β-mercaptoethanol (aMResco, Solon, OH, USA), and 0.0016% (*w*/*v*) bromophenol blue) was mingled with the protein lysate. Electrophoresis at 100 V using 10% (*w*/*v*) SDS-PAGE-separated appropriate amounts of the protein lysates, and the resolved proteins were further transferred onto PVDF membranes (Stratagene, La Jolla, CA, USA). After blocking for 1 h in 3% (*w*/*v*) bovine albumin serum (BSA) (aMResco, Solon, OH, USA) at room temperature, the membranes were hybridized with primary antibodies for 2 h at room temperature. Then, the membranes were washed with TTBS buffer [0.5 M NaCl, 0.02 M Tris-HCl, 0.05% (*v*/*v*) Tween-20] for 15 min four times, and the bound primary antibodies were probed with appropriate secondary antibodies for 1 h at room temperature. The chemiluminescence ECL detection system (GE Healthcare Bio-Sciences AB, Uppsala, Sweden) perceived secondary antibodies binding on the membranes by implementing the Fujifilm LAS-4000 Luminescent Image Analyzer (Fujifilm Corporation, Tokyo, Japan). The pixels of each protein strip, corrected with the actin, were measured by PDQUEST Quantity One software (Bio-Rad Laboratory, Hercules, CA, USA) and evaluated by Student’s *t*-Test (STATISTICA Ver 10.0 MR1, StatSoft, Tulsa, OK, USA).

### 2.8. Immunohistochemical Staining

T24 cell-xenografted tumors were induced in mice, as described by Chang et al. [14]. In brief, animal examinations were carried out under the guidelines of the Laboratory Animal Facilities and Care, as announced by the Council of Agriculture, Executive Yuan, Taiwan., which obeys the Declaration of Helsinki. The practice was agreed by the Institutional Animal Care and Use Committee (IACUC) (permit number: MED-100-05; project code: NSC 101-2632-B-218-001-MY3) of Southern Taiwan University of Science and Technology. We bought male nude mice (BALB/cAnN-Foxn1, nine weeks old) from the National Science Council animal center in Taiwan. Mice were fostered under 30–70% humidity and at 23–25 °C with a 12-h bright/12-h night alternating cycle. The mice ate ad libitum germ-free water and rodent Lab Diet 5001 (Lab Supply, Fort Worth, TX, USA). We quarantined the mice for seven days prior to the experiment. We subcutaneously (s.c.) injected the mice on the right lower abdomen with a 100 µL T24 cell/matrigel combination created by mingling 1 × 107 of T24 cells with an identical volume of the gel. We subsided the discomfort of the mice with all our efforts. The indicated dried EtOAc extract was mixed thoroughly in water. The subsequent suspension was exploited for feeding the mice oral gavage (o.g.). We divided the mice into four subgroups. The xenografted mice were supplied o.g. with water (n = 11), 5 mg/kg (n = 10), 10 mg/kg (n = 10), and 100 mg/kg (n = 6) of EtOAc extract. After being grafted with T24 cells, the extract-treated mice were fed with EtOAc extract suspension by o.g. on the following day. Then, the mice were supplied with the suspension once a day until the endpoint. At the endpoint, each mouse was given intraperitoneally (i.p.) 0.5 mL of 500 mg/mL urethane for euthanization, and the tumor and liver were gathered. Vernier calipers measured the development of the xenografted tumors at 3-day intermissions. The tumor volume was computed as V = length × width2/2. The results are indicated as the mean ± standard error (SE) for the statistical analyses of at least three animals. Statistical significance was determined by Student’s *t*-test and one-way ANOVA using the SigmaPlot program for Windows, version 12.0 (Systat Software Inc., San Jose, CA, USA).

### 2.9. Immunohistochemical Staining

Paraffin-embedded tumor sections (5 μm thick) were hybridized with primary antibodies against HSP90 (1:250, #ab13492, Abcam) and PTP1B (1:250, #ab59373, Abcam). Afterward, the slides were reacted with the secondary antibody (Dako Envision + System-HRP Labelled Polymer, Anti-Rabbit) for 1 h. Slides were developed using 3,3′-diaminobenzidine substrate-chromogen solution (Dako). The hematoxylin was counter-stained in T24 xenografted bladder tumors per mouse. The specimens were quantified using image analysis software (AxioVision; Zeiss, Oberkochen, Germany). The score scale considers the intensity and percentage of positively stained cells. The expression of HSP90 and PTP1B in the tumor biopsies was scored based on the following scale from 0 to 5—0 (negative, no staining), 1 (greater than 1 up to 20% of positive cells, weak intensity), 2 (21–40% of positive cells, weak to moderate intensity), 3 (41–60% of positive cells, moderate to strong intensity), 4 (61–80% of positive cells and strong intensity) and 5 (>80% of positive cells and strong intensity). CFL and LYT blindly evaluated all the specimens.

## 3. Results

### 3.1. Two-Dimensional Gel Electrophoresis of PEPE2-Treated T24 Cells

**Figure 1:**
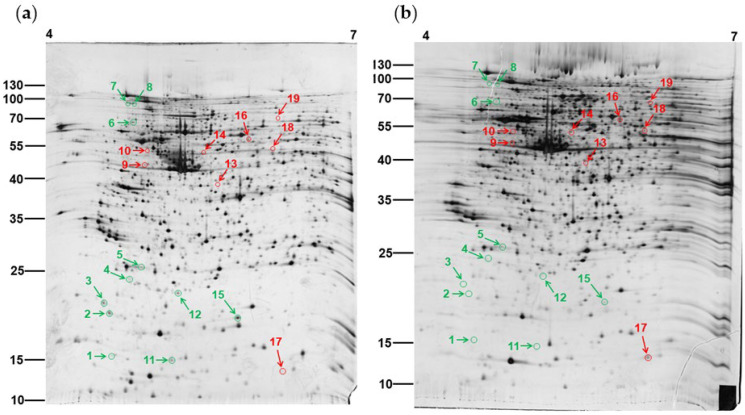
The proteomes of human T24 cells. (**a**) Un-treated cells. (**b**) PEPE2-treated T24 cells. T24 cells were incubated with 20 μg/mL of PEPE2 for 24 h, and the untreated cells were T24 cells incubated with DMSO (the vehicle for PEPE2). A red arrow indicated the upregulated proteins, and a green arrow displayed the downregulated proteins. The original blots see Figure S2.

Our previous studies showed that PEPE2 treatment could inhibit UBUC cells through an apoptotic mechanism. This study further explored the molecular mechanism that originated apoptosis in UBUC cells. Silver-stained two-dimensional gel electrophoresis (2-DE) gels coupled with LC-MS/MS were carried out to obtain protein expression profiles and find those proteins whose amounts were changed by PEPE2 in UBUC cells. Since PEPE2 incubation indicated a better inhibition effect on T24 cell growth [14], T24 cells were exploited for the proteomics study. T24 cells incubated with or without 20 μg/mL PEPE2 for 24 h were chosen for 2-DE analyses because a sufficient number of impacted cells that were still alive could be obtained, and possibly pertinent cellular changes could be acquired before cell death could be observed in PEPE2-treated T24 cells. Firstly, 100 μg of lysates from naïve and PEPE2-exposed T24 cells were clarified using 2-DE gels of 18-cm gel strips (pI 4–7). Two replicate gel pairs were acquired from each of the three independent examinations to reduce the gel-to-gel variation. Figure 1 demonstrated the typical 2-DE gel profile of un-exposed and PEPE2-treated T24 cells, and a supplementary file provided all six gel pairs. As described in Materials and Methods, comparing the proteomic profiles of PEE-administrated T24 cells to those of unexposed cells identified the protein spot differences, and then the differentially expressed proteins were determined. The comparison results indicated that eleven downregulated and eight overexpressed proteins were recognized as represented by the arrowed spots in Figure 1. Table S1 in the Supplementary Materials demonstrated the total normalized volume (ppm) and the statistical result of each protein spot. All the differentially expressed protein spots were statistically significant (*p* < 0.05), except spot 8, which was near significant (*p* = 0.0824).

### 3.2. Recognition of the Differentially Expressed Proteins in PEPE2-Influenced T24 Cells

After the proteome comparison, dysregulated proteins were discovered using LC-MS/MS, as described in Materials and Methods. Table 1 indicated the findings of the spectrometric analyses and protein identification. The pI and experimental molecular weight of each protein spot were close to the theoretical values, and almost all of the spectrometric protein coverages were more than 20%. All the spots were over the 2-fold difference. Table S2 showed the corresponding peptides of each de-regulated protein. Ontology data of Ingenuity pathway analysis (IPA) implicated that the unveiled dysregulated proteins were involved in carbohydrate metabolism, cell apoptosis, cell proliferation, oxidation of fatty acid, ATP metabolism, death receptor signaling, eNOS signaling, JAK/STAT signaling, PPAR pathway, PPARα/RXR α pathway, Rho family GTPase signaling, and RhoGDI signaling (Table 2 and Figure S1).

### 3.3. Validation of Dysregulated Proteins

**Figure 2:**
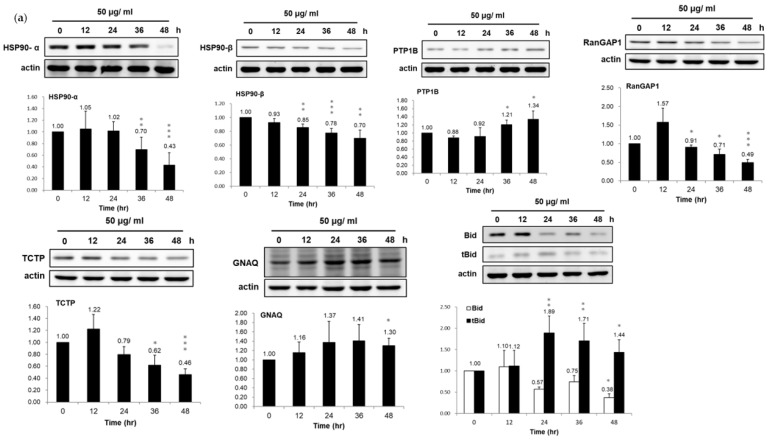

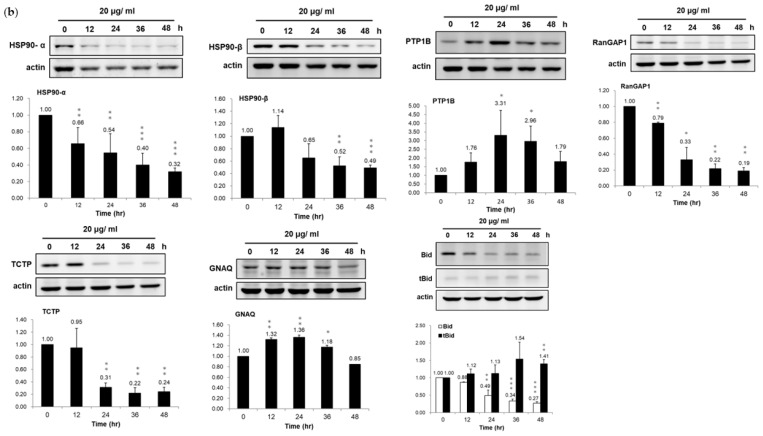
Confirmation of the expression of seven de-regulated proteins in PEPE2-treated. UBUC cells. (**a**) PEPE2-treated T24 cells. (**b**) PEPE2-incubated J82 cells. The original blots see Figure S2. *, **, and *** denote *p* less than 0.05, 0.01, and 0.001 respectively.

Among the PEPE2-induced deregulated proteins, heat shock protein (HSP) 90-α, HSP 90-β, protein-tyrosine phosphatase 1B (PTP1B), Ran GTPase-activating protein 1 (RanGAP1), human translationally controlled tumor protein (TCPT), Guanine nucleotide-binding protein G(q) subunit α (GNAQ), and Bid were chosen to validate our observation on the proteomics results using western immunoblotting. These seven proteins were targeted attributable to their respective high-fold changes and likely linked to cell proliferation and apoptosis. In line with the proteomics results, the data of western immunoblotting demonstrated that HSP 90-α, HSP 90-β, RanGAP1, TCTP, and Bid were under-expressed while PTP1B and GNAQ were over-expressed, respectively, in the PEPE2-treated T24 and J82 cells (Figure 2). Meanwhile, corresponding to the downregulation of Bid, the active form of t-Bid was up-regulated in PEPE2-incubated T24 and J82 cells, which can evoke apoptosis.

**Figure 3:**
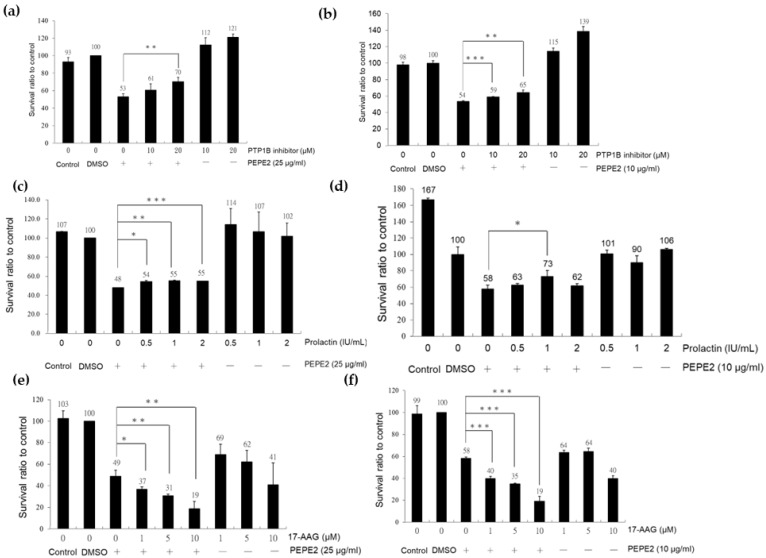
Treatment of UBUC cells with PTP1B inhibitor, prolactin, and 17-AAG. (**a**) T24 cells with PTP1B inhibitor. (**b**) J82 cells with PTP1B inhibitor. (**c**) T24 with prolactin. (**d**) J82 cells with prolactin. (**e**) T24 cells with 17-AAG; (**f**) J82 cells with 17-AAG. *, **, and *** denote *p* less than 0.05, 0.01, and 0.001 respectively.

To further confirm the involvement of PTP1B and HSP90 in PEPE2-evoked apoptosis, we investigated the impacts of PTP1B inhibitor, prolactin (an HSP90 agonist), and 17-327 allylamino-17-demethoxygeldanamycin (17-AAG) (an HSP90 inhibitor) on PEPE2-exposed J82 and T24 cells. The J82 and T24 cells’ survival was restored with the increment amount of PTP1B inhibitor under the influence of PEPE2, while the PTP1B inhibitor alone did not affect the cell survival (Figure 3a,b). The prolactin treatment minorly ameliorated the T24 and J82 cells’ existence in the presence of PEPE2 (Figure 3c,d). On the other end, the coincubation of 17-AAG and PEPE2 provoked much stronger apoptotic effects than 17-AAG alone (Figure 3e,f). The above observation implicated that PTP1B and HSP90 were undoubtedly associated with the apoptotic process evoked by PEPE2.

### 3.4. Association of HSP90/Akt/ASK-1/JNK Pathway with Bladder Cancer Cell Apoptosis

**Figure 4:**
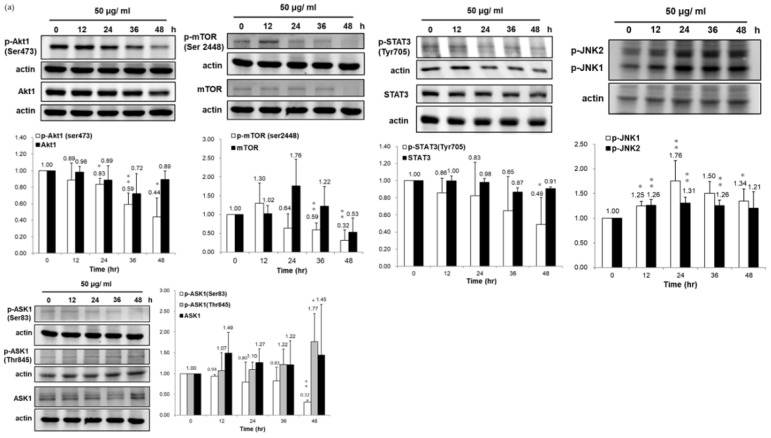

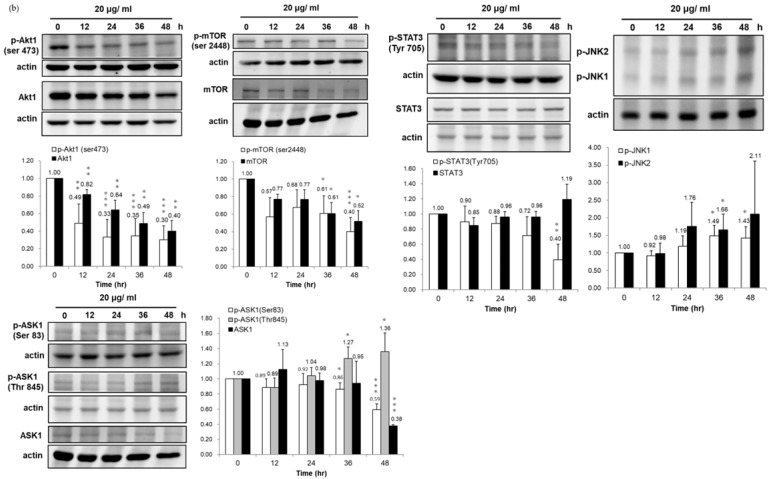
The effects of PEPE2 on the HSP90/Akt/ASK-1/JNK pathway in bladder cancer cells. (**a**) T24 cells. (**b**) J82 cells. The original blots see Figure S2. *, **, and *** denote *p* less than 0.05, 0.01, and 0.001 respectively.

Hsp90 is an essential chaperon for conformational integrity and the activities of some oncogenic transcription factors and signaling effector proteins [17], such as protein kinases (Akt and Erk), signal transducer, and activator of transcription-3 (STAT3), epidermal growth factor receptor (EGFR), and insulin-like growth factor receptor (IGF-R), hypoxia-inducible-factor-1α (HIF-1α). Also, IPA indicated that HSP90 participates in the STAT pathway. Thus, we examined the influences of PEPE2 on the expression of client proteins of HSP90 and the Akt/mTOR/STAT3 pathway. Our data presented that PEPE2 incubation profoundly inhibited Akt-1 phosphorylation in the T24 and J82 cells, while the treatment barely affected the Akt-1 expression (Figure 4). Following the Akt-1 results, PEPE2′s presence meaningfully curbed mTOR phosphorylation and profoundly decreased the phosphorylated STAT3 (active form) expression with no effect on STAT3 expression in the T24 and J82 cells. STAT3 is one of the client proteins of mTOR [18]. The reduced p-STAT3 amount was possibly attributable to overexpressed PTP1B (Figure 4). Surprisingly, PEPE2 treatment significantly inhibited mTOR synthesis in T24 cells at a 48-h duration. However, the treatment dwindled mTOR expression in J82 cells at every duration, especially at the 36- and 48-h periods. In addition to Akt-1, HSP90 phosphorylates ASK-1 at ser83 via Akt-1 and inhibits Ask-evoked apoptosis [19]. Figure 4 demonstrated that ser83 phosphorylation of Ask-1 decreased due to reduced HSP90 expression provoked by PEPE2 administration in the T24 and J82 cells, which initiated apoptosis. In contrast, Thr845 phosphorylation of ASK-1 induces p53-mediated apoptosis [20]. Our results indicated that PEPE2 incubation triggered the increment of Thr845 phosphorylation of ASK-1. In addition, Wang et al. reported that HSP90 inhibition decreases pSer83 but increases pThr845 and p38/JNK activation. Consistent with Wang’s observation, PEPE2 therapy could upregulate the phosphorylated JNK expression in T24 and J82 cells. The earlier findings implicated that PEPE2 might evoke apoptosis by regulating the HSP90/Akt/ASK-1/JNK pathway in bladder cancer cells.

### 3.5. The Validation of PTPB1 and HSP90α Expression in Xenografted Bladder Tumor Mice

**Figure 5:**
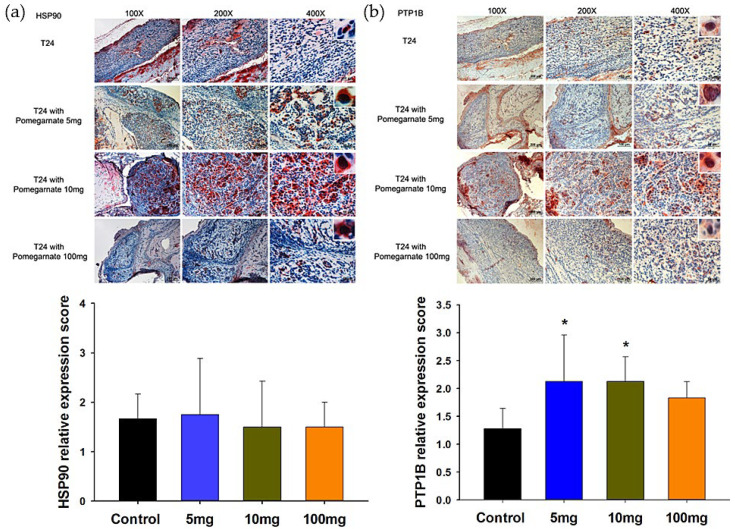
The influences of PEPE2 on the expression of HSP90 and PTP1B in mice T24 xenografted bladder tumors. (**a**) HSP90. (**b**) PTP1B. * denotes *p* less than 0.05.

Our previous xenografted bladder cancer studies [14] found that PEPE2 treatment could evoke tumor cell apoptosis. This study evaluated PTP1B and HSP90-α levels in PEPE2-incubated xenografted bladder cancer mice using IHC to confirm the expression of PTP1B and HSP90α. We observed that the PTP1B expression significantly increased in PEPE2-cured xenografted mice compared to mock-treated tumor mice, while the HSP90-α level decreased slightly in a dose-dependent manner (Figure 5). The above findings were in line with those of proteomics studies.

## 4. Discussion

Our previous observations showed that the PEPE2 fraction exhibited the best inhibitory activities among the eight fractions from column chromatography of the ethanol extract’s EtOAc layer from pomegranate peel. In this study, we implemented 2-DE gel coupled with tandem mass spectrometry to uncover the molecular mechanism of the inhibitory activities of the PEPE2 fraction toward the UBUC cells to find the target proteins of the PEPE2 fraction. The results of gel-based proteomics indicated that 20 μg/mL PEPE2 incubation induced eleven underexpressed and eight overexpressed proteins in T24 cells. The proteomics and validation data aligned with the IPA-predicted and acknowledged functions of deregulated proteins. IPA analyses showed that PTP1B, GNAQ, SOD1, BID, FABP4, HSP90-α, HSP90-β, TCTP, and DPYSL3 were involved in cell apoptosis. FABP4 intimately participates in bladder cancer cell progression, and Bid is well-known for the pro-apoptotic protein. Our results demonstrated that FABP4 and Bid were downregulated, while the t-Bid (active form) was increased in the PEPE2-treated UBUC cells. However, the IHC results displayed that PEPE2 feeding only slightly decreased HSP90-α protein amount in the tumor compared to that of the untreated control, while PEPE2 treatment significantly lowered the HSP90-α expression in the PEPE2-cultured T24 and J82 UBUC cells. This discrepancy might be attributed to the difference between gene signatures of cell lines and tumor tissue. Moreover, the quantitative evaluation of Western immunoblotting is different from that of IHC.

The HSP90 serves as a molecular chaperone to maintain the integrity, folding, and function of several essential transcription factors and kinases associated with apoptosis, proliferation, survival, DNA damage, and repairs, such as NF-κB, p53, JNK, Akt, Erk, EGFR, IGF-R, Raf-1 [17]. The IPA search data implicated that HSP90 and PTP1B participate in the JAK/STAT3 pathway. (Table 2) [21]. In line with the previously documented results and IPA prediction, our discoveries established that the PEPE2 remedy decreased the expression of HSP-90-α and HSP90-β and thereby reduced the expression of phospho-Akt, phospho-mTOR (the client protein of Akt), and phosphor-STAT3 (the downstream effector of mTOR) in UBUC cells. The Akt-1/mTOR/STAT3 pathway is essential for cell proliferation. Zhang et al. reported that HSP90 could phosphorylate ASK-1 at ser83 through Akt-1 and mitigates Ask-1-evoked apoptosis [19], while Thr845 phosphorylation of ASK-1 induces p53-mediated apoptosis [20]. Our results confirmed that PEPE2 treatment increased the Thr845 phosphorylation of ASK-1 and downregulated the ser83 phosphorylation in UBUC cells, suggesting that the PEPE2 cure likely intervented the HSP90/Akt-1/Ask-1 pathway to induce tumor cell apoptosis. Many documented observations report that PTP1B can negatively regulate the JAK/STAT3 pathway that regulates cell proliferation [22,23,24]. Some studies demonstrate that PTP1B might act as a tumor suppressor in NSCLC, esophageal cancer, and lymphoma [25,26,27]. The proteomics and confirmation results demonstrated that PEPE2 treatment could increase PTP1B expression, and thus, the JAK/STAT3 pathway was inhibited in bladder cancer cells, implying that PTP1B might be a tumor suppressor protein in UBUC. The role of PTP1B in the tumorigenesis of bladder cancer remains to be elucidated in the future. The above observations implicated that PEPE2 therapy might disturb the HSP90/Akt-1/mTOR/STAT3 and PTP1B/STAT3 pathways to inhibit cancer cell proliferation. Also, PEPE2 treatment might interfere with the HSP90/Akt-1/ASK-1 cascade to provoke apoptosis in the bladder cancer cell.

Our findings showed that PEPE2 incubation could decrease the TCTP amount in bladder cancer, thus matching the findings that TCTP serves as an anti-apoptotic protein and is linked to the mTOR pathway and cell cycle regulation [28]. Many studies found that GNAQ mutation prevails in uveal melanoma [29,30]. GNAQ signaling leads to the activation of the oncoprotein YAP that regulates the Hippo pathway along with oncoprotein TAZ. The impaired Hippo pathway for organ size control may contribute to various tumors [31,32]. GNAQ mediates the YAP activation through Trio, a guanine nucleotide exchange factor, and its downstream target GTPases Rho and Rac, well-known for regulating the actin cytoskeleton [33]. The proteomics and IPA blast search data illustrated that PEPE2 therapy could deregulate the GNAQ expression, and GNAQ was associated with Rac/Rho pathway and cytoskeleton assembly. In line with the abovementioned findings, this study’s results suggested that PEPE2 treatment might inhibit bladder cancer cell proliferation by manipulating the cytoskeleton assembly through dysregulating GNAQ expression. In addition to the proteins controlling cancer cell proliferation and apoptosis, our proteomics results found that PEPE2 administration could interfere with DPYSL3 expression in cancer cells, which is involved in cell adhesion and the metastasis of various tumors, such as breast cancer [34], lung cancer [35], gastric cancer [36], and prostate cancer [37].

Nowadays, many cancer therapies focus on the intervention of tumor cells’ metabolic mechanisms. The IPA analyses in this study demonstrated that PEPE2 therapy is connected with ATP metabolism and the PPAR pathway essential for controlling cellular metabolism (Table 2) [38], thus indicating that the PEPE2 remedy might curb bladder cancer cell survival because of the energy metabolism disturbance.

## 5. Conclusions

This study uncovered a more detailed molecular mechanism underlying the pomegranate. We disclosed that PEPE2 fraction from the ethyl acetate extract of pomegranate could inhibit the bladder tumor by regulating (1) the HSP90-associated pathway, which regulates the cell proliferation; (2) PTP1B, which is a tumor suppressor; (3) apoptotic genes; and (4) cytoskeleton assembly.

## Figures and Tables

**Table 1:** The identities of the deregulated proteins in PEPE2-treated T24 cells.

Spot	Protein Identity	Incidences	Experiment PI/MW	Theoretical PI/MW	Matched Peptide Number	Coverage (%) ^1^	Accession Number (NCBI)	Fold
1	myosin light polypeptide 6 (MYL6)	6/6	4.6/16.8	4.56/17	3	43.1	119617305	−8.9
2	myosin regulatory light chain MRLC2 (MYL2)	6/6	4.6/20.6	4.67/19.7	7	48.84	119622081	−8.1
3	coatomer protein complex, subunit zeta 1 (COPZ1)	5/6	4.6/21.8	4.69/20.2	3	28.81	119617180	−50.7
4	Chromobox protein homolog 3 (CBX3)	5/6	4.8/24.9	5.23/20.8	6	33.33	116241284	−3.7
5	Human Translationally Controlled Tumor Protein (TCTP)	5/6	4.9/26.3	5.24/20.7	3	21.11	114794484	−7.3
6	Ran GTPase-activating protein 1 (RanGAP1)	5/6	4.8/66.7	4.63/63.5	14	30.83	1172922	−4
7	Heat shock protein HSP 90-beta (HSP90-β)	5/6	4.8/82.8	4.96/83.3	10	20.02	17865718	−4.1
8	heat shock protein HSP 90-alpha (HSP90-α)	5/6	4.8/82.7	5.11/77.3	6	9.95	153792590	−2.5
9	vimentin (V.I.M.)	6/6	4.9/44.2	5.41/42.8	13	36.69	119606621	2.6
10	Mitochondrial ATP synthase subunit beta (ATP5B)	5/6	4.9/50.8	5.26/56.6	18	50.28	114549	9.1
11	Coactosin-like Protein-1 (COTL1)	5/6	5.4/16.7	5.5/15.9	7	60.56	119615882	−14.1
12	Pro-Apoptotic Protein Bid (Bid)	5/6	5.4/23.6	5.25/22.1	2	13.7	159163783	−6.1
13	Guanine nucleotide-binding protein alpha-q (GNAQ)	5/6	6.2/40.8	5.48/42.1	6	18.94	251757492	4.6
14	RuvB-like 2; 48 kDa TATA box-binding protein interacting protein (RUVBL2)	5/6	5.9/52.6	5.49/51.2	12	35.2	28201890	3.8
15	Superoxide dismutase 1 (SOD1)	5/6	6.4/21.6	5.7/15.9	4	57.14	134611	−4.9
16	Human Prolidase (PEPD)	5/6	6.5/58.4	5.64/54.6	6	14.57	112491419	2.7
17	Fatty acid-binding protein 4 (FABP4)	5/6	6.6/16.2	6.59/14.7	2	48.48	119781	14.6
18	protein-tyrosine phosphatase 1B (PTP1B)	5/6	6.6/51.4	5.88/50	7	32.87	131467	7.6
19	dihydropyrimidinase-related protein 3 (DPYSL3)	5/6	6.6/71.4	5.94/73.9	10	33.18	308818200	5

^1^ The percentage of matched peptide sequences in an identified protein sequence.

**Table 2:** The functions of differentially expressed proteins ^1^.

Protein Function	Protein ^2^
Carbohydrate metabolism	PTP1B, GNAQ, SOD1, BID, FABP4
Cell apoptosis	PTP1B, GNAQ, SOD1, B.I.D., FABP4, HSP90-α, HSP90-β, TCTP, DPYSL3
Proliferation of cells	DPYSL3, FABP4, GNAQ, HSP90-α, HSP90-β, PTP1B, RUVBL2, SOD1, TCTP, ATP5B, B.I.D., COPZ1
Oxidation of fatty acid	B.I.D., FABP4
Synthesis of ATP	SOD1, ATP5B
Catabolism of ATP	ATP5B, RUVBL2
Death receptor signaling	BID
eNOS signaling	HSP90-α, HSP90-β, GNAQ
JAK/STAT signaling	PTP1B, GNAQ, HSP90-α, HSP90-β
PPAR pathway	HSP90-α, HSP90-β, PTP1B
PPARα/R.X.R. α pathway	HSP90-α, HSP90-β,GNAQ
Rho family GTPase signaling	GNAQ, MYL2
RhoGDI signaling	GNAQ, MYL2

^1^ The protein functions were predicted using Ingenuity pathway analysis. ^2^ Protein abbreviation: PTP1B, Protein-tyrosine phosphatase 1B, GNAQ, Guanine nucleotide-binding protein G(q) subunit α, SOD1, Superoxide dismutase 1, B.I.D., Pro-Apoptotic Protein Bid, FABP4, Fatty acid-binding protein 4, HSP90, Heat shock protein 90, TCTP, human translationally controlled tumor protein, DPYSL3, Dihydropyrimidinase-related protein 3, RUVBL2, RuvB-like 2, ATP5B, Mitochondrial ATP synthase subunit β, COPZ1, Coatomer protein complex, subunitζ1, MYL2, Myosin regulatory light chain.

## Data Availability

Not applicable.

## Supplementary Materials

The following supporting information can be downloaded at: https://www.mdpi.com/article/10.3390/life12111839/s1, Figure S1: The IPA results; Figure S2: The original blots of Figure 1, Figure 2a,b and Figure 4a,b; Table S1: The total normalized volume and the statistical result of each protein spot; Table S2: The matched peptides of each de-regulated protein; Antibodies; Collection and Identification of Plant Materials; Preparation of the ethanol extracts from pomegranate peels.

## References

[1] Siegel R.L., Miller K.D., Fuchs H.E., Jemal A. (2021). Cancer Statistics, 2021. CA Cancer J. Clin..

[2] (2018). Health Promotion Administration, Ministry of Health and Welfare, Taiwan, Cancer Registry Annual Report. https://www.hpa.gov.tw/Pages/Detail.aspx?nodeid=269&pid=13498.

[3] Zieger K., Wolf H., Olsen P., Højgaard K. (2001). Long-term follow-up of noninvasive bladder tumours(stage Ta): Recurrence and progression. Br. J. Urol..

[4] Sharma P., McClees S.F., Afaq F. (2017). Pomegranate for Prevention and Treatment of Cancer: An Update. Molecules.

[5] Khan N., Hadi N., Afaq F., Syed D.N., Kweon M.-H., Mukhtar H. (2007). Pomegranate fruit extract inhibits prosurvival pathways in human A549 lung carcinoma cells and tumor growth in athymic nude mice. Carcinogenesis.

[6] Khan N., Afaq F., Kweon M.-H., Kim K., Mukhtar H. (2007). Oral Consumption of Pomegranate Fruit Extract Inhibits Growth and Progression of Primary Lung Tumors in Mice. Cancer Res..

[7] Malik A., Afaq F., Sarfaraz S., Adhami V.M., Syed D.N., Mukhtar H. (2005). Pomegranate fruit juice for chemoprevention and chemotherapy of prostate cancer. Proc. Natl. Acad. Sci. USA.

[8] Adhami V.M., Siddiqui I.A., Syed D.N., Lall R.K., Mukhtar H. (2011). Oral infusion of pomegranate fruit extract inhibits prostate carcinogenesis in the TRAMP model. Carcinogenesis.

[9] Lee S.-T., Wu Y.-L., Chien L.-H., Chen S.-T., Tzeng Y.-K., Wu T.-F. (2012). Proteomic exploration of the impacts of pomegranate fruit juice on the global gene expression of prostate cancer cell. Proteomics.

[10] Koyama S., Cobb L.J., Mehta H.H., Seeram N.P., Heber D., Pantuck A.J., Cohen P. (2010). Pomegranate extract induces apoptosis in human prostate cancer cells by modulation of the IGF–IGFBP axis. Growth Horm. IGF Res..

[11] Rettig M.B., Heber D., An J., Seeram N.P., Rao J.Y., Liu H., Klatte T., Belldegrun A., Moro A., Henning S.M. (2008). Pomegranate extract inhibits androgen-independent prostate cancer growth through a nuclear factor-κB-dependent mechanism. Mol. Cancer Ther..

[12] Pantuck A.J., Leppert J.T., Zomorodian N., Aronson W., Hong J., Barnard R.J., Seeram N., Liker H., Wang H., Elashoff R. (2006). Phase II Study of Pomegranate Juice for Men with Rising Prostate-Specific Antigen following Surgery or Radiation for Prostate Cancer. Clin. Cancer Res..

[13] Pantuck A.J., Pettaway C.A., Dreicer R., Corman J.M., Katz A., Ho A., Aronson W.J., Clark W., Simmons G.W., Heber D. (2015). A randomized, double-blind, placebo-controlled study of the effects of pomegranate extract on rising PSA levels in men following primary therapy for prostate cancer. Prostate Cancer Prostatic Dis..

[14] Chang C.-P., Chan Y.-Y., Li C.-F., Chien L.-H., Lee S.-T., Wu T.-F. (2018). Deciphering the Molecular Mechanism Underlying the Inhibitory Efficacy of Taiwanese Local Pomegranate Peels against Urinary Bladder Urothelial Carcinoma. Nutrients.

[15] Sheng K.-H., Yao Y.-C., Chuang S.-S., Wu H., Wu T.-F. (2006). Search for the tumor-related proteins of transition cell carcinoma in Taiwan by proteomic analysis. Proteomics.

[16] Li C.-F., Shen K.-H., Chien L.-H., Huang C.-H., Wu T.-F., He H.-L. (2018). Proteomic Identification of the Galectin-1-Involved Molecular Pathways in Urinary Bladder Urothelial Carcinoma. Int. J. Mol. Sci..

[17] Lanneau D., Brunet M., Frisan E., Solary E., Fontenay M., Garrido C. (2008). Heat shock proteins: Essential proteins for apoptosis regulation. J. Cell. Mol. Med..

[18] Zhou J., Wulfkuhle J., Zhang H., Gu P., Yang Y., Deng J., Margolick J.B., Liotta L.A., Petricoin E., Zhang Y. (2007). Activation of the PTEN/mTOR/STAT3 pathway in breast cancer stem-like cells is required for viability and maintenance. Proc. Natl. Acad. Sci. USA.

[19] Zhang R., Luo D., Miao R., Bai L., Ge Q., Sessa W.C., Min W. (2005). Hsp90–Akt phosphorylates ASK1 and inhibits ASK1-mediated apoptosis. Oncogene.

[20] Madan E., Gogna R., Kuppusamy P., Bhatt M., Mahdi A.A., Pati U. (2013). SCO2 Induces p53-Mediated Apoptosis by Thr ^845^ Phosphorylation of ASK-1 and Dissociation of the ASK-1–Trx Complex. Mol. Cell. Biol..

[21] Kolosenko I., Grander D., Tamm K. (2014). IL-6 activated JAK/STAT3 pathway and sensitivity to Hsp90 inhibitors in multiple myeloma. Curr. Med. Chem..

[22] Tsunekawa T., Banno R., Mizoguchi A., Sugiyama M., Tominaga T., Onoue T., Hagiwara D., Ito Y., Iwama S., Goto M. (2017). Deficiency of PTP1B Attenuates Hypothalamic Inflammation via Activation of the JAK2-STAT3 Pathway in Microglia. eBioMedicine.

[23] Lee Y.-J., Song H., Yoon Y.J., Park S.-J., Kim S.-Y., Han D.C., Kwon B.-M. (2020). Ethacrynic acid inhibits STAT3 activity through the modulation of SHP2 and PTP1B tyrosine phosphatases in DU145 prostate carcinoma cells. Biochem. Pharmacol..

[24] Lund I.K., Hansen J.A., Andersen H.S., Møller N.P.H., Billestrup N. (2005). Mechanism of protein tyrosine phosphatase 1B-mediated inhibition of leptin signalling. J. Mol. Endocrinol..

[25] Warabi M., Nemoto T., Ohashi K., Kitagawa M., Hirokawa K. (2000). Expression of Protein Tyrosine Phosphatases and Its Significance in Esophageal Cancer. Exp. Mol. Pathol..

[26] Dubé N., Bourdeau A., Heinonen K.M., Cheng A., Loy A.L., Tremblay M.L. (2005). Genetic Ablation of Protein Tyrosine Phosphatase 1B Accelerates Lymphomagenesis of p53-Null Mice through the Regulation of B-Cell Development. Cancer Res..

[27] Martínez-Meza S., Díaz J., Sandoval-Bórquez A., Valenzuela-Valderrama M., Díaz-Valdivia N., Rojas-Celis V., Contreras P., Huilcaman R., Ocaranza M.P., Chiong M. (2019). AT2 Receptor Mediated Activation of the Tyrosine Phosphatase PTP1B Blocks Caveolin-1 Enhanced Migration, Invasion and Metastasis of Cancer Cells. Cancers.

[28] Koziol M.J., Gurdon J.B. (2012). TCTP in Development and Cancer. Biochem. Res. Int..

[29] Schadendorf D., Fisher D.E., Garbe C., Gershenwald J.E., Grob J.-J., Halpern A., Herlyn M., Marchetti M.A., McArthur G., Ribas A. (2015). Melanoma. Nat. Rev. Dis. Prim..

[30] Chattopadhyay C., Kim D.W., Gombos D.S., Oba J., Qin Y., Williams M.D., Esmaeli B., Grimm E.A., Wargo J.A., Woodman S.E. (2016). Uveal melanoma: From diagnosis to treatment and the science in between. Cancer.

[31] Overholtzer M., Zhang J., Smolen G.A., Muir B., Li W., Sgroi D.C., Deng C.-X., Brugge J.S., Haber D.A. (2006). Transforming properties of *YAP*, a candidate oncogene on the chromosome 11q22 amplicon. Proc. Natl. Acad. Sci. USA.

[32] Dong J., Feldmann G., Huang J., Wu S., Zhang N., Comerford S.A., Gayyed M.F., Anders R.A., Maitra A., Pan D. (2007). Elucidation of a Universal Size-Control Mechanism in *Drosophila* and Mammals. Cell.

[33] Nobes C.D., Hall A. (1995). Rho, Rac, and Cdc42 GTPases regulate the assembly of multimolecular focal complexes associated with actin stress fibers, lamellipodia, and filopodia. Cell.

[34] Matsunuma R., Chan D.W., Kim B.-J., Singh P., Han A., Saltzman A.B., Cheng C., Lei J.T., Wang J., da Silva L.R. (2018). DPYSL3 modulates mitosis, migration, and epithelial-to-mesenchymal transition in claudin-low breast cancer. Proc. Natl. Acad. Sci. USA.

[35] Yang Y., Jiang Y., Xie D., Liu M., Song N., Zhu J., Fan J., Zhu C. (2018). Inhibition of cell-adhesion protein DPYSL3 promotes metastasis of lung cancer. Respir. Res..

[36] Kanda M., Nomoto S., Oya H., Shimizu D., Takami H., Hibino S., Hashimoto R., Kobayashi D., Tanaka C., Yamada S. (2014). Dihydropyrimidinase-like 3 facilitates malignant behavior of gastric cancer. J. Exp. Clin. Cancer Res..

[37] Li B., Li C. (2017). Suppression of Prostate Cancer Metastasis by DPYSL3-Targeted saRNA. RNA Act..

[38] Wagner K.-D., Wagner N. (2010). Peroxisome proliferator-activated receptor beta/delta (PPARβ/δ) acts as regulator of metabolism linked to multiple cellular functions. Pharmacol. Ther..

