# Supplementary material for: Uncovering the Inhibitory Molecular Mechanism of Pomegranate Peel to Urinary Bladder Urothelial Carcinoma Using Proteomics Techniques
Source: Life (Basel). 2022 Nov 9;12(11):1839. doi: 10.3390/life12111839 (PMC9694692; doi:10.3390/life12111839)
Supplement: Supplementary file 1 [file life-12-01839-s001.zip › Supplementary Materials/Supplementary file.pdf]

Antibodies

TCTP (1:10000, Abcam #ab133568, Waltham, MA USA), GNAQ (1:1000, St John's laboratory # STJ43766, London, UK), Bid (1:1000, Abcam #ab32060, Waltham, MA USA), tBid (1:1000, Abcam #ab108293, Waltham, MA USA), β-Actin (1:5000, Millipore #MAB1501, Darmstadt, Germany), Anti-rabbit IgG (1:10000, GE Healthcare #NA934, Wauwatosa, WI, USA), Anti-mouse IgG (1:10000, GE Healthcare #N931, Wauwatosa, WI, USA), Phospho-ASK1 (Ser83) (1:1000, Cell Signaling #3761, St. Louis, MO, USA), Phospho-ASK1 (Thr845) (1:1000, Cell Signaling #3765, St. Louis, MO, USA), ASK1 (1:2000, Abcam #ab45178, Waltham, MA USA), Phospho-STAT3 (Tyr705) (1:10000, Abcam #ab76315, Waltham, MA USA), STAT3 (1:1000, Abcam #ab109085, Waltham, MA USA), Phospho-JNK (T183/Y185) (1:1000, Signaling #4671, St. Louis, MO, USA)

Collection and Identification of Plant Materials

Please refer to Nutrients 2018, 10, 543.

Preparation of the Ethanol Extracts from Pulps and Peels of Pomegranates

Please refer to Nutrients 2018, 10, 543.

Figure S1: The IPA results

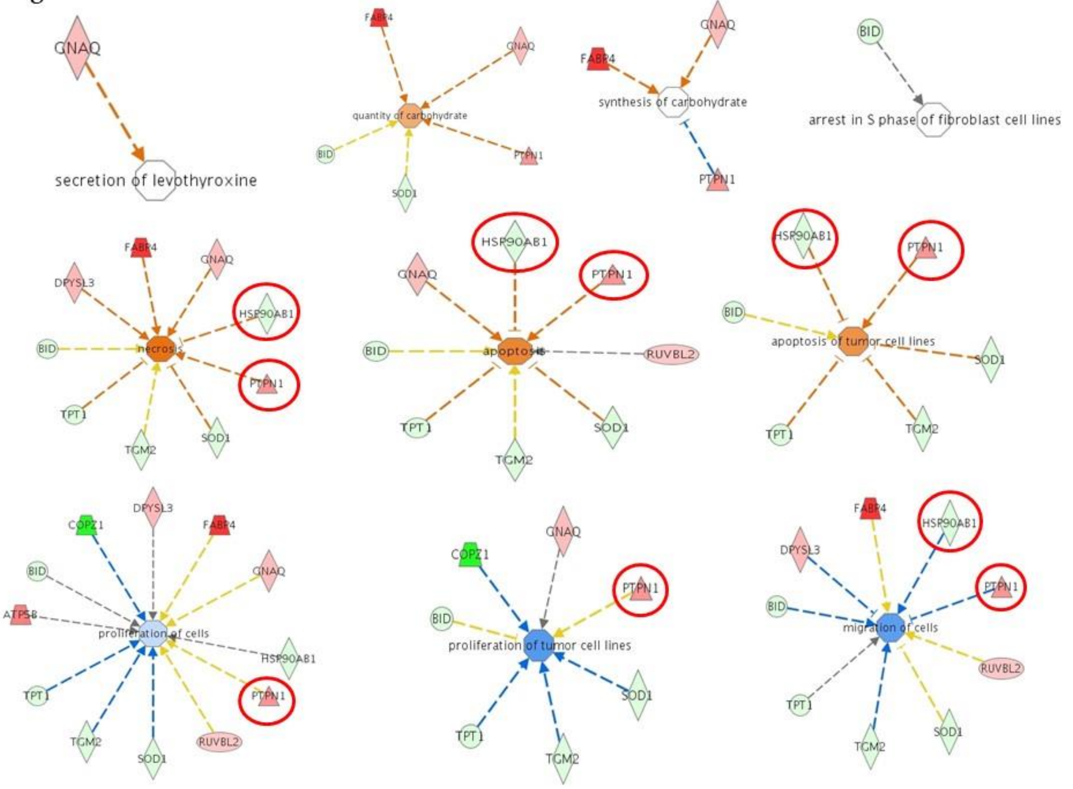

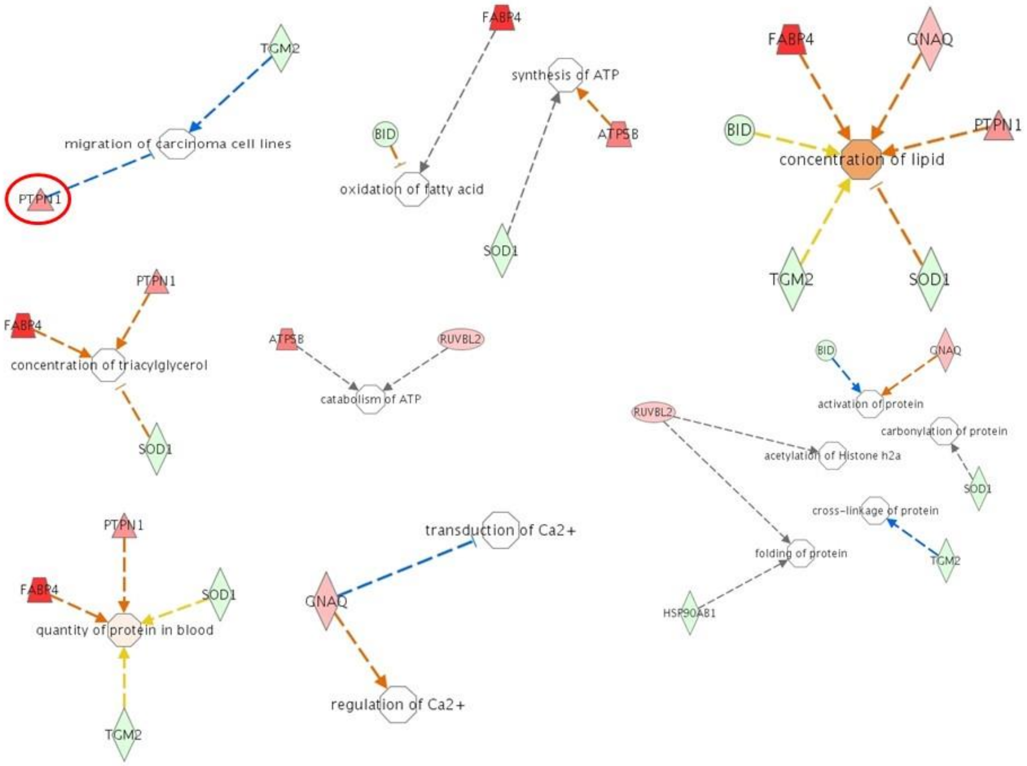
TGM2
migration of carcinoma cell lines
PTPN1
FABP4
BID
oxidation of fatty acid
synthesis of ATP
ATP5B
SOD1
FABP4
GNAQ
BID
PTPN1
concentration of lipid
TGM2
SOD1
PTPN1
FABP4
concentration of triacylglycerol
SOD1
ATP5B
RUVBL2
catabolism of ATP
BID
GNAQ
activation of protein
RUVBL2
carbonylation of protein
acetylation of Histone h2a
SOD1
cross-linkage of protein
folding of protein
TGM2
HSP90AB1
PTPN1
FABP4
SOD1
quantity of protein in blood
TGM2
transduction of Ca2+
GNAQ
regulation of Ca2+

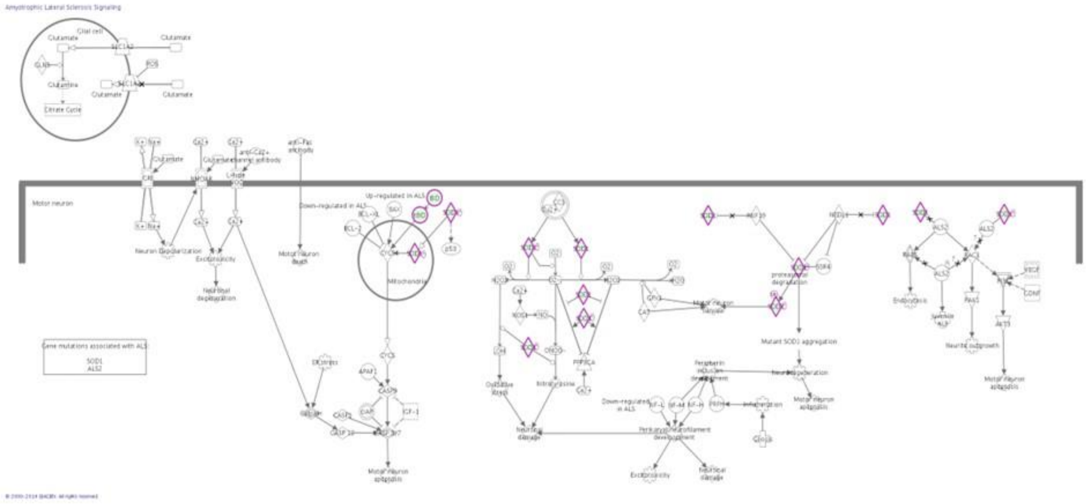
Amyotrophic Lateral Sclerosis Signaling
Motor neuron
Mitochondria
Cene mutations associated with ALS
SOD1
ALS2

Death Receptor Signaling

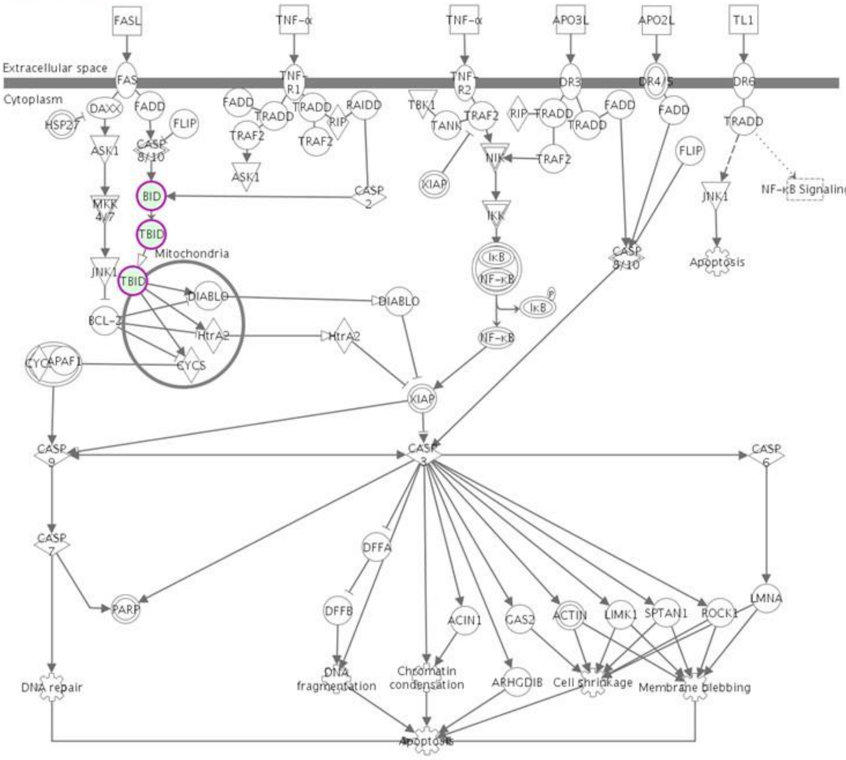

© 2000-2014 QIAGEN. All rights reserved.

eNOS Signaling

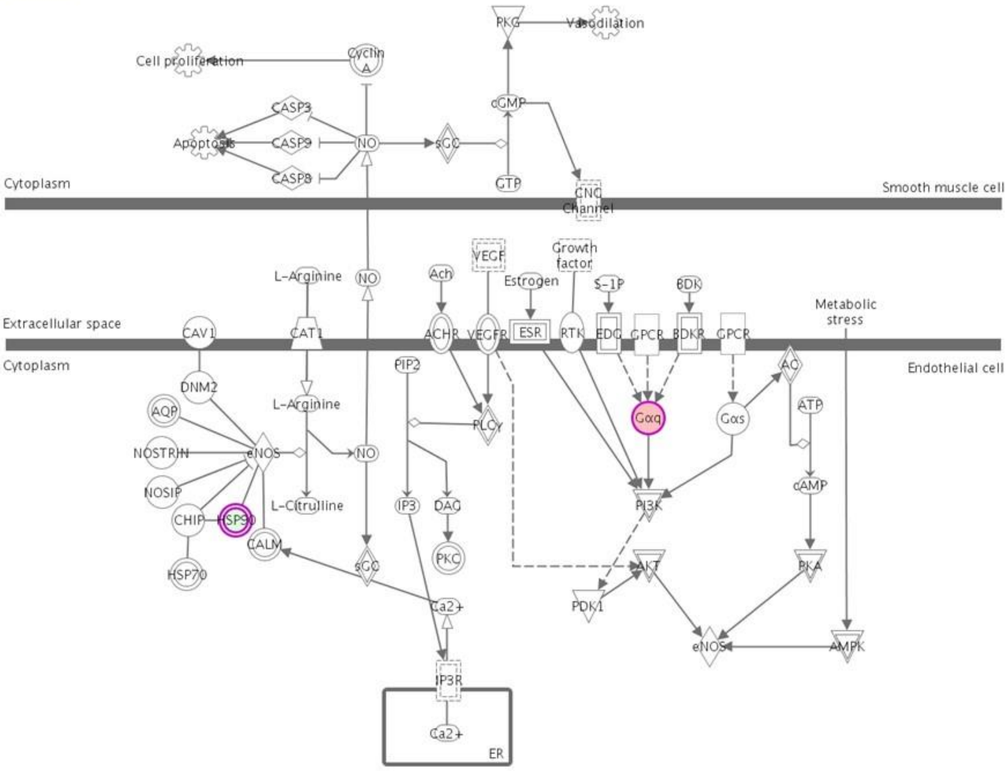

© 2000-2014 QIAGEN. All rights reserved.

JAK/Stat Signaling

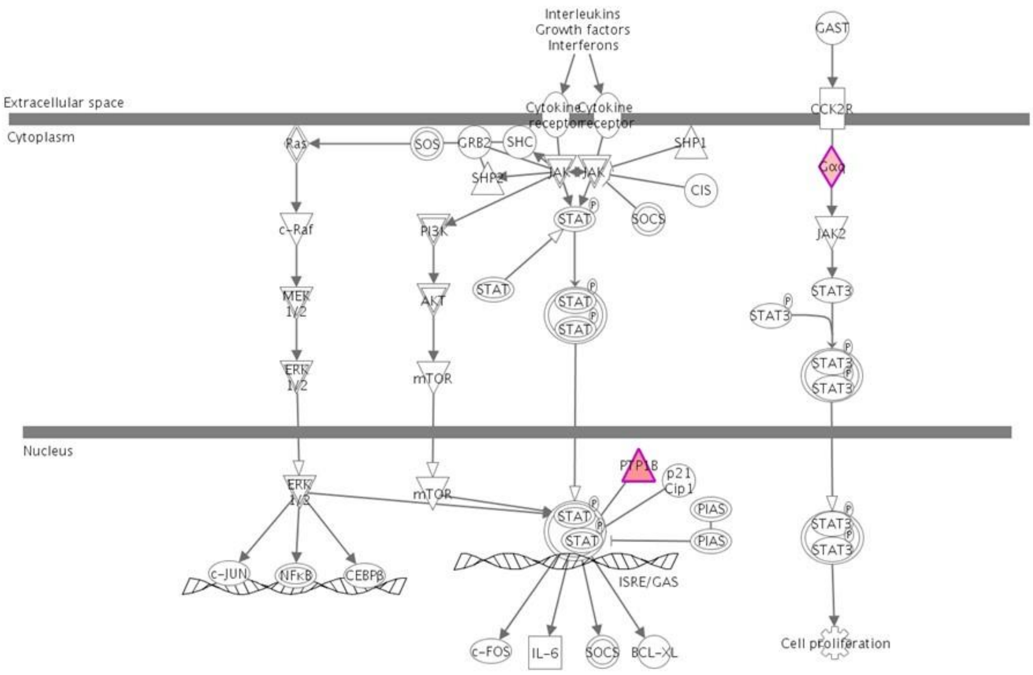

© 2000-2014 QIAGEN. All rights reserved.

PPAR Signaling

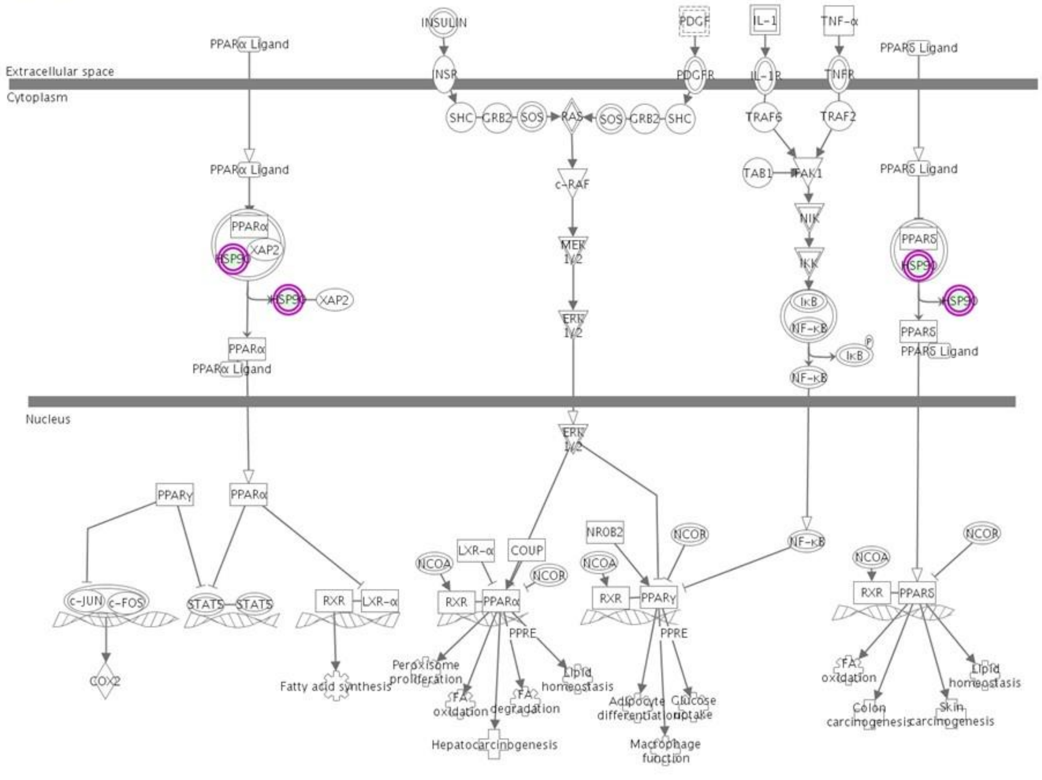

© 2000-2014 QIAGEN. All rights reserved.

PPARα/RXRα Activation

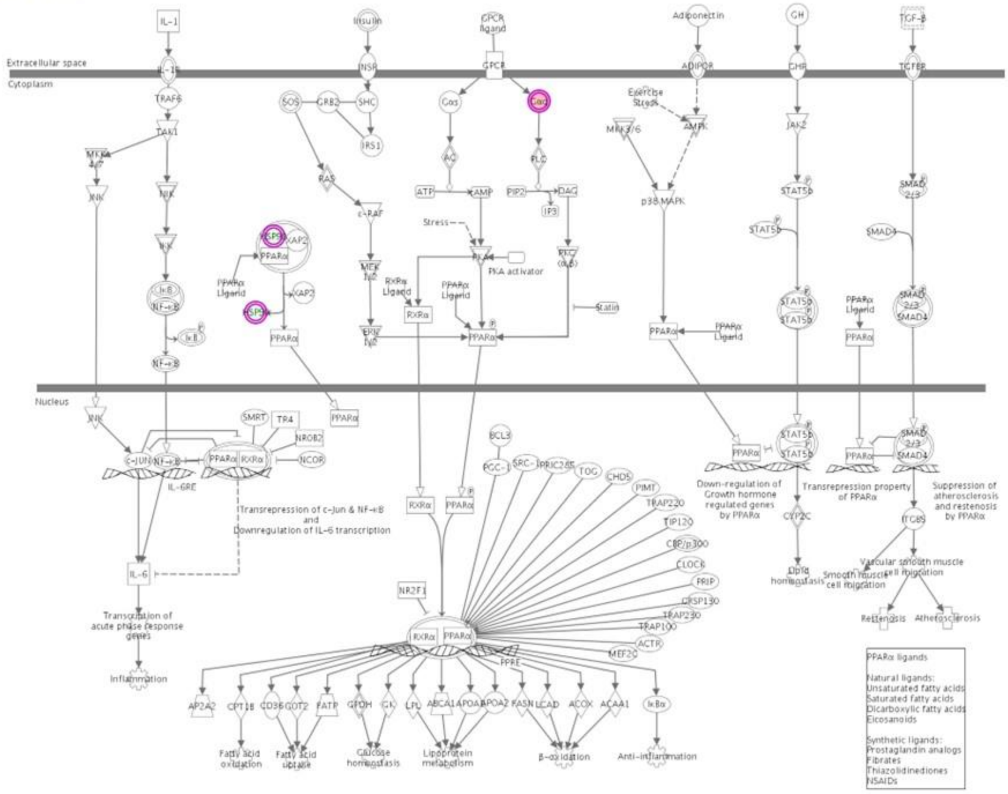

© 2000-2014 QIAGEN. All rights reserved.

Prostate Cancer Signaling

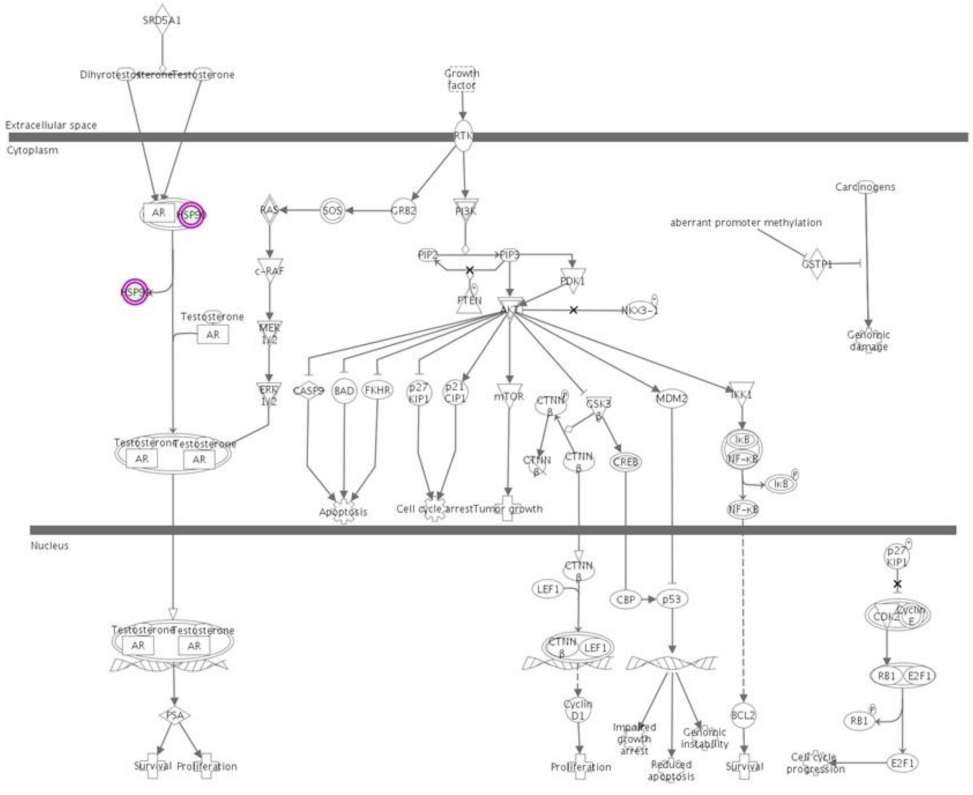

© 2000-2014 QIAGEN. All rights reserved.

Signaling by Rho Family GTPases

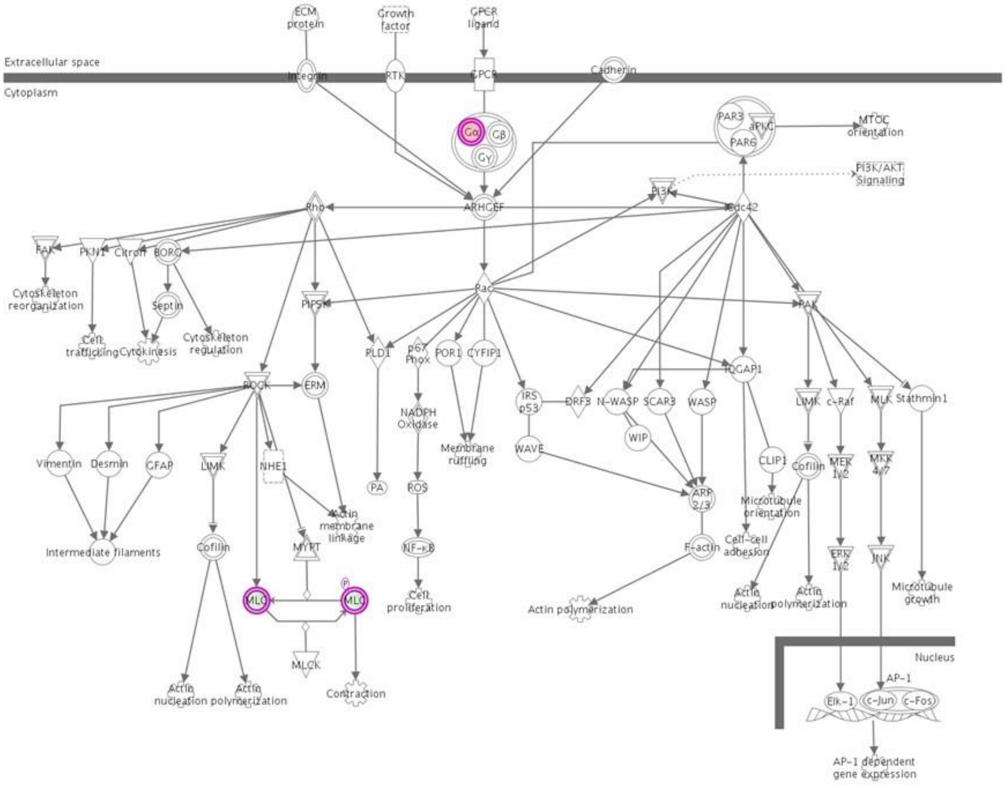

© 2000-2014 QIAGEN. All rights reserved.

RhoGDI Signaling

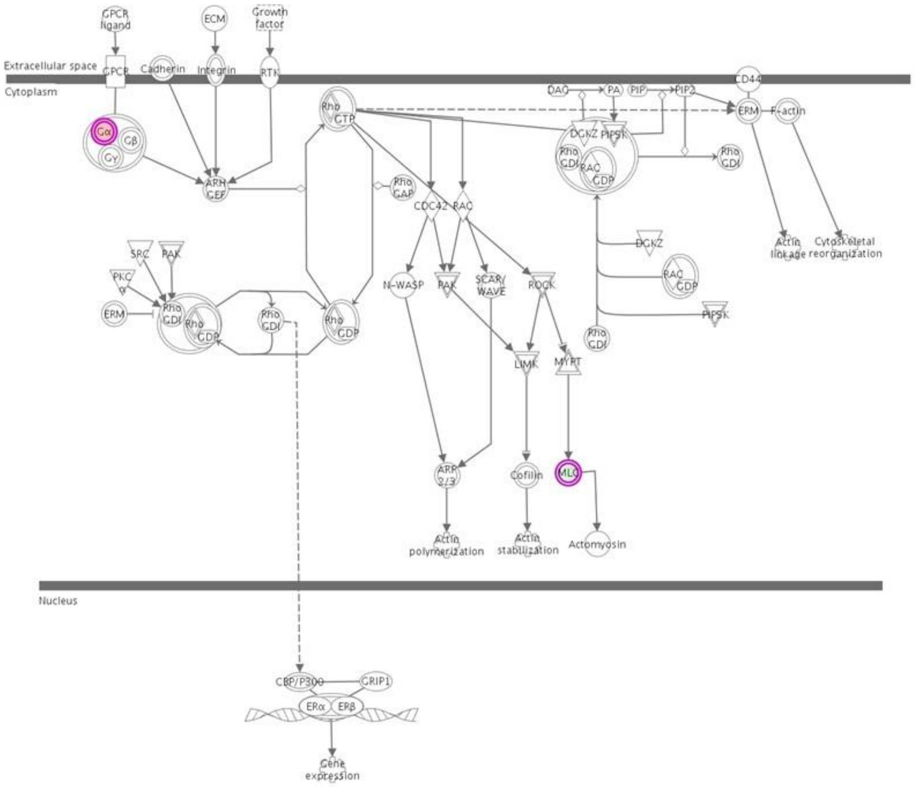

© 2000-2014 QIAGEN. All rights reserved.

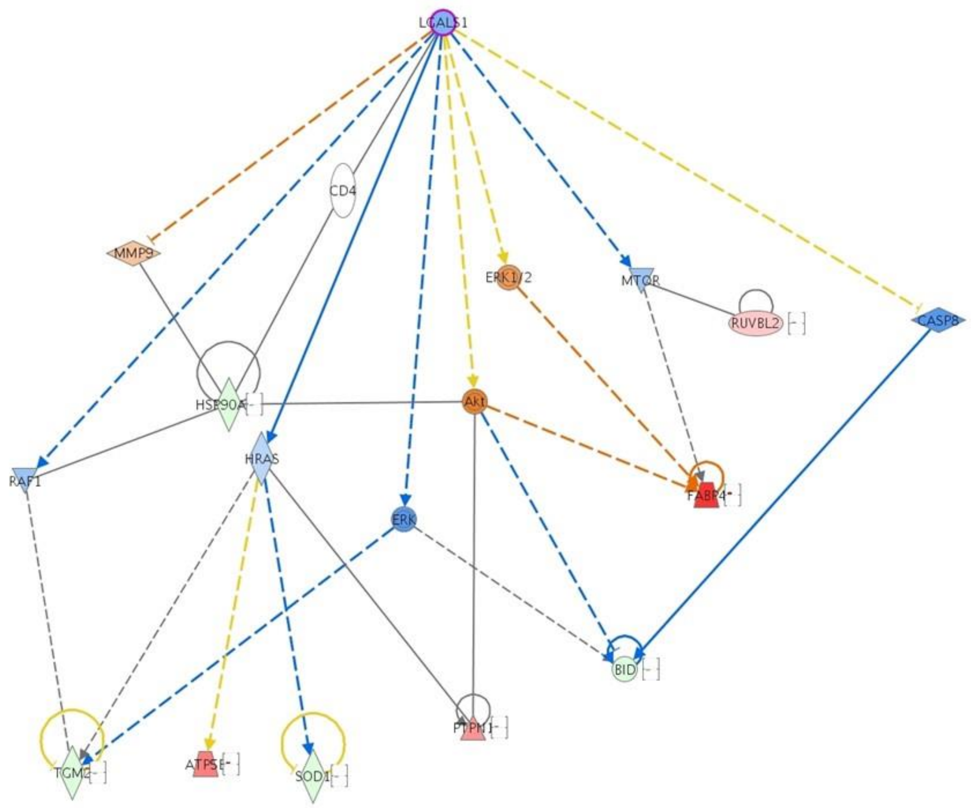

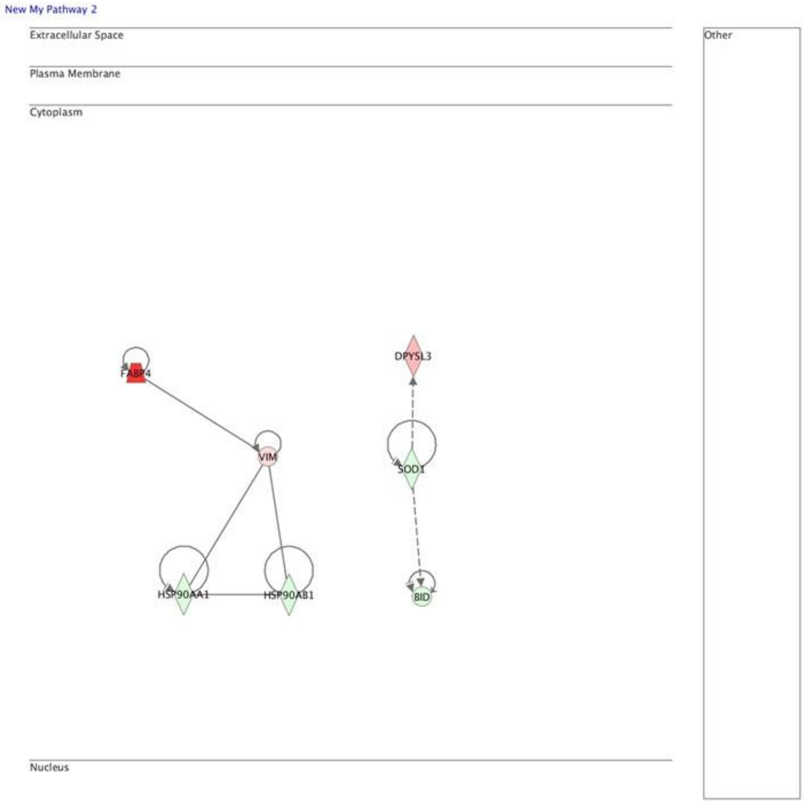

© 2000-2014 QIAGEN. All rights reserved.

Path Designer New My Pathway 4

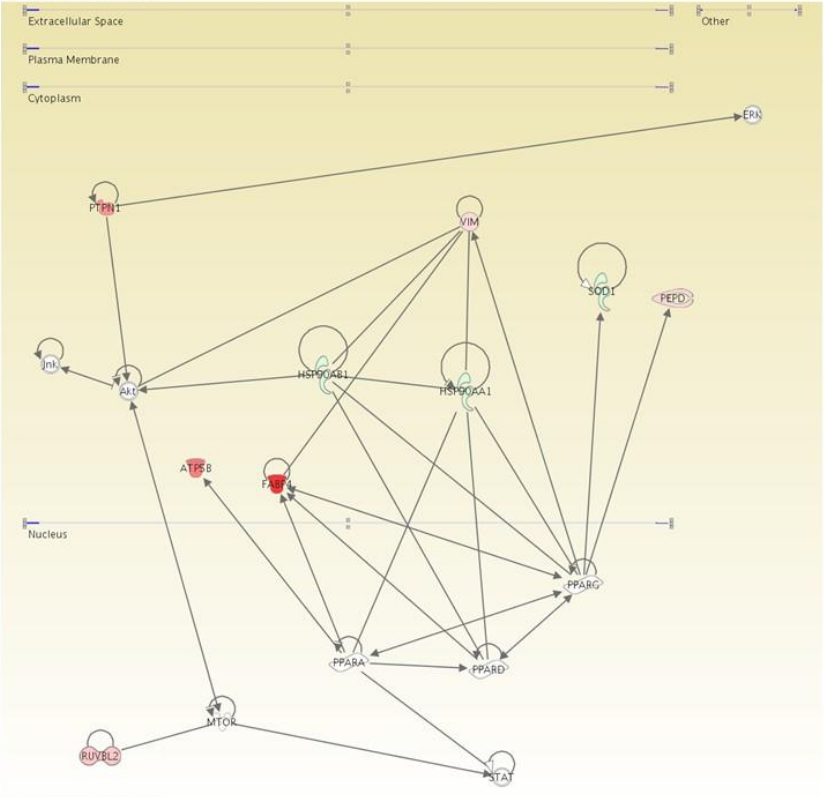

© 2000-2014 QIAGEN. All rights reserved.

Path Designer JAK/Stat Signaling

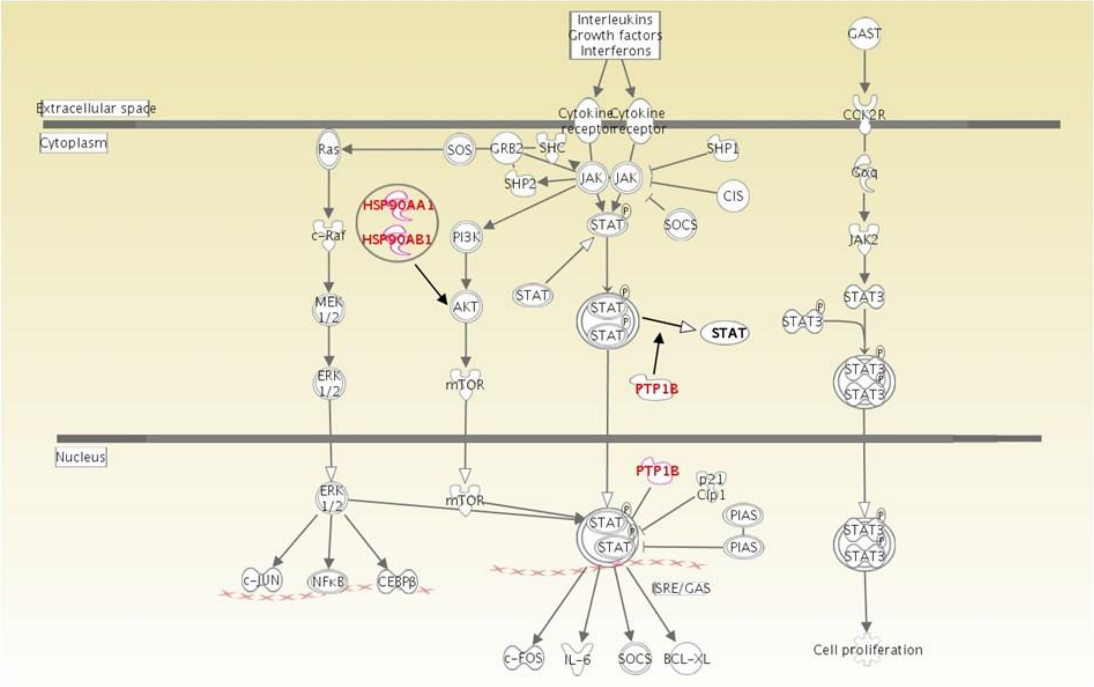

© 2000-2014 QIAGEN. All rights reserved.

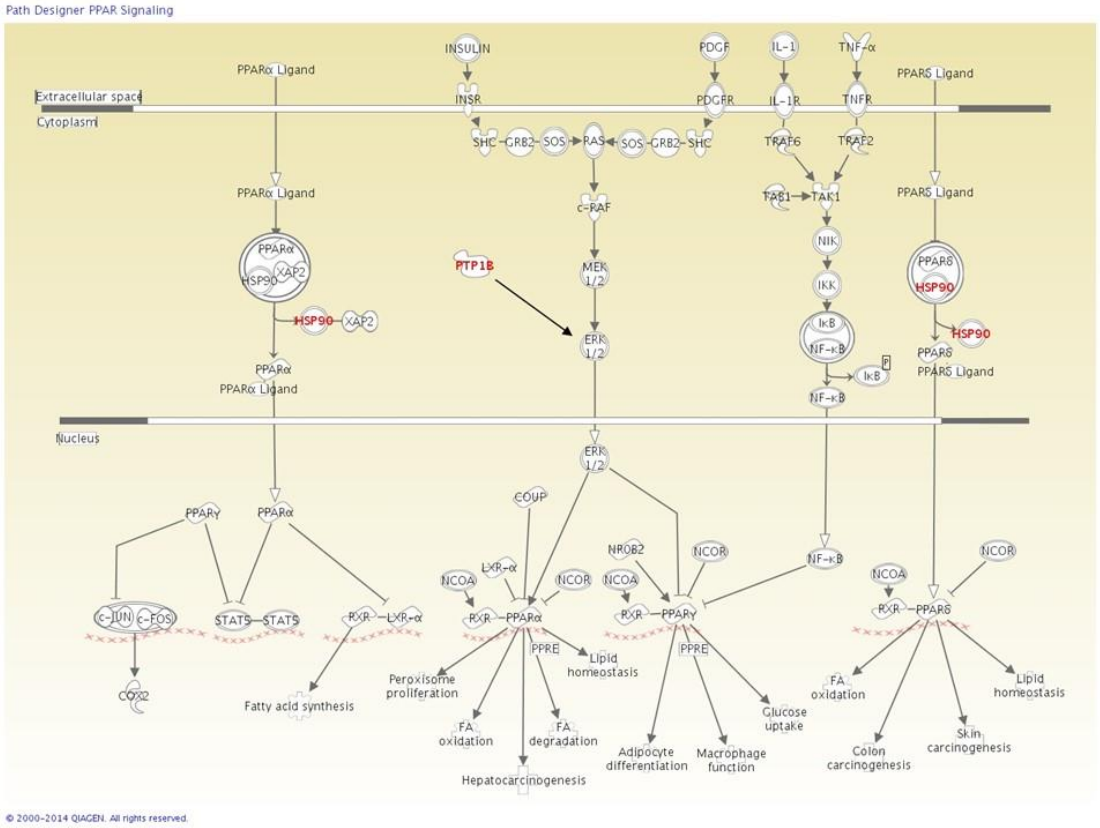

Table S1. The volumes of each de-regulated protein spot.

Spot number	Control (ppm±S.D.)	Treatment (ppm±S.D.)	***P* value**
Spot 1	260.25±145.48	29.27±12.12	0.0153
Spot 2	421.65±85.72	51.90±32.67	0.0024
Spot 3	316.60±71.50	6.24±2.28	0.0025
Spot 4	221.94±41.62	59.60±19.99	0.0079
Spot 5	669.22±131.03	91.52±21.28	0.0024
Spot 6	138.46±50.49	34.42±11.12	0.0465
Spot 7	229.62±58.18	55.38±15.82	0.0202
Spot 8	255.98±70.35	102.12±32.49	0.0824
Spot 9	102.73±14.71	263.52±40.56	0.0039
Spot 10	12.20±4.33	111.22±20.80	0.0016
Spot 11	187.06±92.95	13.26±9.61	0.0086

Spot 12	242.74±73.76	38.22±9.02	0.0250
Spot 13	9.92±2.71	46.00±8.50	0.0037
Spot 14	43.64±9.03	165.88±25.06	0.0018
Spot 15	586.52±196.74	119.22±25.01	0.0462
Spot 16	95.30±19.74	252.78±45.11	0.0126
Spot 17	28.56±8.79	416.14±63.14	0.0003
Spot 18	19.58±7.99	148.18±30.13	0.0033
Spot 19	30.38±13.21	152.36±38.50	0.0172

Table S2. The detailed mass spectrometry data of matched peptides

Spot	Protein I.D.	Matched[2] peptide number	Sequence[1]
1	myosin light polypeptide 6 (MYL6)	3	NKDQGTYEDYVEGLR (2,4.12,0.48,1)<br>ALGQNPTNAEVLK (2,3.28,0.31,1)<br>VLGNPKSDEMNVK (2,3.18,0.32,1)
2	myosin regulatory light chain MRLC2 (MYL2)	7	ATSNVFAMFDQSQIQEFK (2,6.33,0.57,1)<br>FTDEEVDELYREAPIDKK (3,3.90,0.14,1)<br>ELLTTMGDRFTDEEVDELYREAPIDK (3,3.43,0.43,3)<br>KGNFNYIEFTR (2,3.31,0.36,1)<br>GNFNYIEFTR (2,3.26,0.16,1)<br>DGFIDKEDLHDMLASLGK (3,3.06,0.40,2)<br>LNGTDPEDVIR (2,2.77,0.18,1)
3	coatomer protein complex, subunit zeta 1 (COPZ1)	3	VALRGEDVPLTEQTVSQVLQSAK (3,5.53,0.54,1)<br>AILILDNDGDRLFAK (2,4.28,0.54,1)<br>YYDDTYPSVKEQK (2,2.89,0.24,4)
4	Chromobox protein homolog 3 (CBX3)	6	KVEEAEPEEFVVEKVLDR (3,5.40,0.55,1)<br>IIGATDSSGELMFLMK (2,5.32,0.65,1)<br>WKDSDEADLVLAK (3,3.89,0.36,1)<br>SKKVEEAEPEEFVVEK (2,3.60,0.40,1)<br>DSDEADLVLAK (2,3.22,0.36,1)<br>LTWHSCPEDEAQ (2,2.66,0.31,1)
5	Human Translationally Controlled Tumor Protein (TCTP)	3	MIIYRDLISHDEMFSDIYK (3,3.37,0.13,2)<br>DLISHDEMFSDIYKIR (3,2.96,0.42,1)<br>YIKDYMK (2,2.18,0.22,1)
6	Ran GTPase-activating protein 1 (RanGAP1)	14	SSVLIAQQTDTSDPEKVVSAFLK (3,5.19,0.59,1)<br>MAVQDAVDALMQK (2,4.95,0.46,1)

			DAALAVAEAMADKAELEK (2,4.76,0.55,1)<br>NRLENDGATALAEAFR (2,4.51,0.48,1)<br>VINLNDNTFTEK (2,4.15,0.47,1)<br>HSLLQTLYKV (2,3.7,0.48,1)<br>VINLNDNTFTEKGAVAMAETLK (3,3.48,0.33,1)<br>SKGAVAIADAIR (3,3.31,0.44,1)<br>EIEDFDSLEALRLEGNTVGVEAAR (3,3.25,0.26,1)<br>TQVAGGQLSFK (2,3.16,0.44,2)<br>KSSAQGKPLALK (2,3.03,0.34,2)<br>VSSVFKDEATVR (2,2.73,0.41,1)<br>SSAQGKPLALK (2,2.64,0.32,4)<br>GAVAMAETLKTLR (2,2.51,0.38,2)
7	Heat shock protein HSP 90-beta (HSP90-β)	10	NPDDITQEEYGEFYK (2,5.63,0.58,1)<br>HLEINPDHPIVETLR (2,4.43,0.54,1)<br>GVVDSEDLPLNISR (2,4.03,0.4,1)<br>YHTSQSGDEMTSLSEYVSR (3,3.87,0.56,1)<br>ELISNASDALDKIR (2,3.57,0.52,1)<br>ADLINNLGTIAK (2,3.42,0.46,1)<br>VILHLKEDQTEYLEER (3,3.27,0.44,1)<br>TLTLVDTGIGMTK (2,3.09,0.55,1)<br>EQVANSAFVER (2,2.83,0.44,3)<br>YIDQEELNKTKPIWTR (3,2.71,0.43,3)
8	Heat shock protein HSP 90-alpha (HSP90-α)	6	KHLEINPDHSIIETLR (4,4.18,0.40,1)<br>HLEINPDHSIIETLR (3,3.65,0.47,1)<br>NPDDITNEEYGEFYK (2,3.54,0.50,1)<br>ELISNSSDALDKIR (2,2.85,0.35,5)<br>DNSTMGYMAAK (2,2.79,0.38,1)<br>LGIHEDSQNR (2,2.64,0.42,1)
9	vimentin (VIM)	13	LQDEIQNMKEEMAR (2,4.23,0.53,1)<br>ILLAELEQLKGQGK (2,4.19,0.39,1)<br>LQEEMLQREEAENTLQSFR (3,4.12,0.50,1)<br>TNEKVELQELNDR (2,3.85,0.43,1)<br>FADLSEAANR (2,3.65,0.43,1)<br>KVESLQEEIAFLK (2,3.48,0.32,1)<br>NLQEAEEWYKSK (2,3.44,0.42,1)<br>NLQEAEEWYK (2,3.40,0.32,1)<br>LGDLYEEEMR (2,3.16,0.30,1)<br>QDVDNASLAR (2,3.11,0.37,1)<br>DGQVINETSQHHDDLE (2,3.04,0.34,2)<br>ISLPLPNFSSLNLR (2,2.56,0.36,1)<br>ETNLDSLPLVDTHSKR (2,2.47,0.43,4)
10	Mitochondrial ATP synthase subunit beta (ATP5B)	18	EGNDLYHEMIESGVINLK (2,5.53,0.51,1)<br>TREGNDLYHEMIESGVINLKDATSK (3,5.26,0.35,1)<br>LVLEVAQHLGESTVR (2,4.99,0.61,1)<br>FTQAGSEVSALLGR (2,4.95,0.53,1)<br>VALVYGQMNEPPGAR (2,4.90,0.57,1)<br>FLSQPFQVAEVFTGHMGK (2,4.85,0.56,1)<br>TVLIMELINNVAK (2,4.73,0.43,1)

			IMDPNIVGSEHYDVAR (2,4.68,0.56,1)<br>SLQDIIAILGMDELSEEDKLTVSR (3,4.57,0.60,1)<br>GQKVLDSGAPIKIPVGPETLGR (3,4.57,0.55,1)<br>AIAELGIYPAVDPLDSTSR (2,4.33,0.58,1)<br>VLDSGAPIKIPVGPETLGR (3,4.25,0.47,1)<br>EGNDLYHEMIESGVINLK (3,4.11,0.17,6)<br>IMNVIGEPIDERGPIKTK (2,3.63,0.46,1)<br>IPSAVGYQPTLATDMGTMQER (3,3.63,0.40,1)<br>IGLFGGAGVGK (2,3.30,0.47,1)<br>ETRLVLEVAQHLGESTVR (3,3.08,0.33,12)<br>TIAM*DGTEGLVR (2,2.32,0.23,2)
11	Coactosin-Like Protein-1 (COTL1)	7	TGTDKTLVKEVVQNFAK (3,5.32,0.61,1)<br>ELEEDFIKSELKK (2,4.26,0.32,1)<br>TLVKEVVQNFAK (2,3.81,0.42,1)<br>SKFALITWIGENVSGLQR (3,3.38,0.34,1)<br>EFVISDRKELEEDFIK (3,3.25,0.11,1)<br>FTTGDAMSKR (2,2.76,0.45,1)<br>AKTGTDKTLVK (2,2.38,0.25,2)
12	Pro-Apoptotic Protein Bid (Bid)	2	LGRIEADSESQEDIIR (3,4.98,0.48,1)<br>HLAQVGDSMDR (2,3.42,0.46,1)
13	Guanine nucleotide-binding protein alpha-q (GNAQ)	6	YYLNDLDRVADPAYLPTQQDVLR (3,6.13,0.47,1)<br>VSAFENPYVDAIK (2,4.29,0.40,1)<br>VADPAYLPTQQDVLR (2,3.78,0.44,1)<br>IIHGSGYSDEDKR (2,3.53,0.1,1)<br>LLLLGTGESGK (2,2.87,0.24,1)<br>RINDEIER (2,2.46,0.15,3)
14	RuvB-like 2；48 kDa TATA box-binding protein interacting protein (RUVBL2)	12	ALESDMAPVLIMATNR (2,4.58,0.58,1)<br>TQGFLALFSGDTGEIKSEVR (3,4.12,0.55,1)<br>IRGTSYQSPHGIPIDLLDR (3,3.48,0.42,1)<br>LLIVSTTPYSEKDTK (2,3.42,0.41,2)<br>DKVQAGDVITIDKATGK (2,3.36,0.60,1)<br>EVVHTVSLHEIDVINSR (3,3.26,0.43,1)<br>GLGLDDALEPR (2,3.26,0.43,1)<br>AVLIAGQPGTGK (2,3.20,0.50,1)<br>QASQGMVGQLAAR (2,3.15,0.46,1)<br>ARDYDAMGSQTK (2,3.01,0.48,1)<br>RAAGVVLEMIR (2,2.94,0.28,16)<br>VYSLFLDESR (2,2.58,0.33,1)
15	Superoxide dismutase 1 (SOD1)	4	TLVVHEKADDLGKGGNEESTK (3,5.69,0.26,1)<br>GDGPVQGIINFEQK (2,4.28,0.50,1)<br>HVGDLGNVTADK (2,3.09,0.50,1)<br>LACGVIGIAQ (2,2.65,0.25,1)
16	Human Prolidase (PEPD)	6	NPAVQAGSIVVLQGGEETQR (2,6.21,0.60,1)<br>YTNKISSEAHR (3,3.81,0.59,1)

			VFKTDMELEVLR (2,3.53,0.47,1)<br>TDMELEVLR (2,3.00,0.38,1)<br>LPASHATWMGK (2,2.94,0.53,1)<br>AVYEAVLR (2,2.04,0.30,5)
17	Fatty acid-binding protein 4 (FABP4)	2	LVSSENFDDYMKEVGVGFATRK (3,4.05,0.56,1)<br>NTEISFILGQEFDEVTADDRK (3,3.92,0.57,1)
18	protein-tyrosine phosphatase 1B (PTP1B)	7	IKLHQEDNDYINASLIKMEEAQR (4,4.66,0.51,1)<br>QLELENLTTQETR (2,4.30,0.27,1)<br>FSYLAVIEGAK (2,4.24,0.51,1)<br>FIMGDSSVQDQWK (2,3.80,0.49,1)<br>GSPLNAAPYGIESMSQDTEVR (3,3.66,0.44,1)<br>SGSWAAIYQDIR (2,3.44,0.39,1)<br>EMIFEDTNLK (2,2.67,0.24,4)
19	dihydropyrimidinase-related protein 3 (DPYSL3)	10	MDENQFVAVTSTNAAK (2,5.27,0.11,1)<br>NLHQSGFSLSGTQVDEGVR (2,5.16,0.64,1)<br>ISVGSDSDLVIWDPDAVK (2,5.02,0.55,1)<br>IMLEDGNLHVTQGAGR (2,4.86,0.59,1)<br>GMYDGPVFDLTTTPK (2,4.12,0.63,1)<br>TLDFDALSVGQR (2,3.90,0.54,1)<br>EESREPAPASPAPAGVEIR (3,3.41,0.20,9)<br>GMTTVDDFFQGTK (2,3.29,0.48,2)<br>SAADLISQAR (2,2.54,0.21,1)<br>IFNLYPR (2,2.41,0.29,4)

[1]The numbers in parentheses correspond to z, Xcorr, dCn, Rsp parameters of TurboSequest respectively.

[2] Matched peptide number: number of peptides matched with protein in MS/MS query.
