# Supplementary figures and images for: Uncovering the Inhibitory Molecular Mechanism of Pomegranate Peel to Urinary Bladder Urothelial Carcinoma Using Proteomics Techniques
Source: Life (Basel). 2022 Nov 9;12(11):1839. doi: 10.3390/life12111839 (PMC9694692; doi:10.3390/life12111839)

Figure S2. Original blots.

Figure 1  
Control

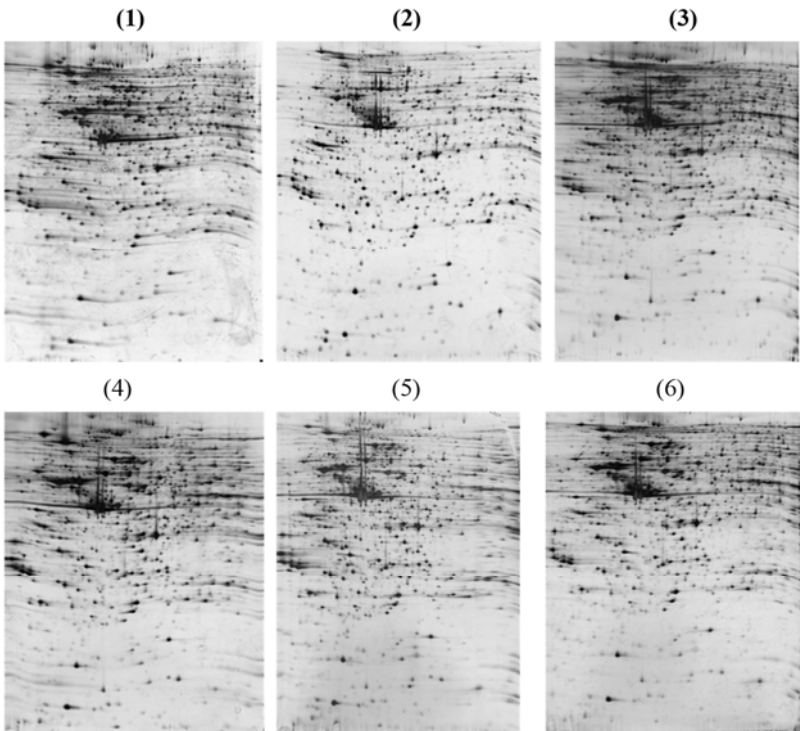

Treatment

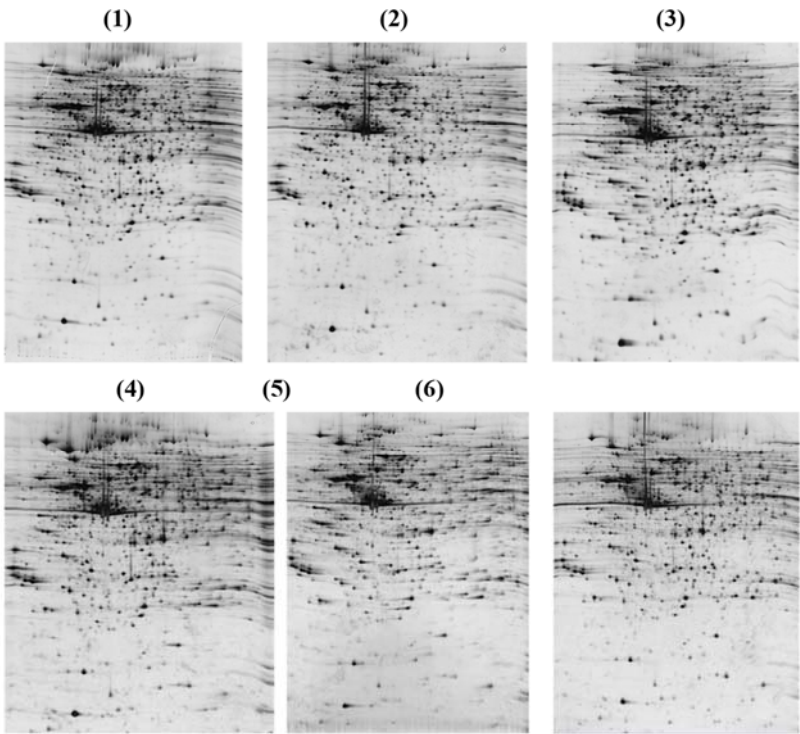

Figure 2a

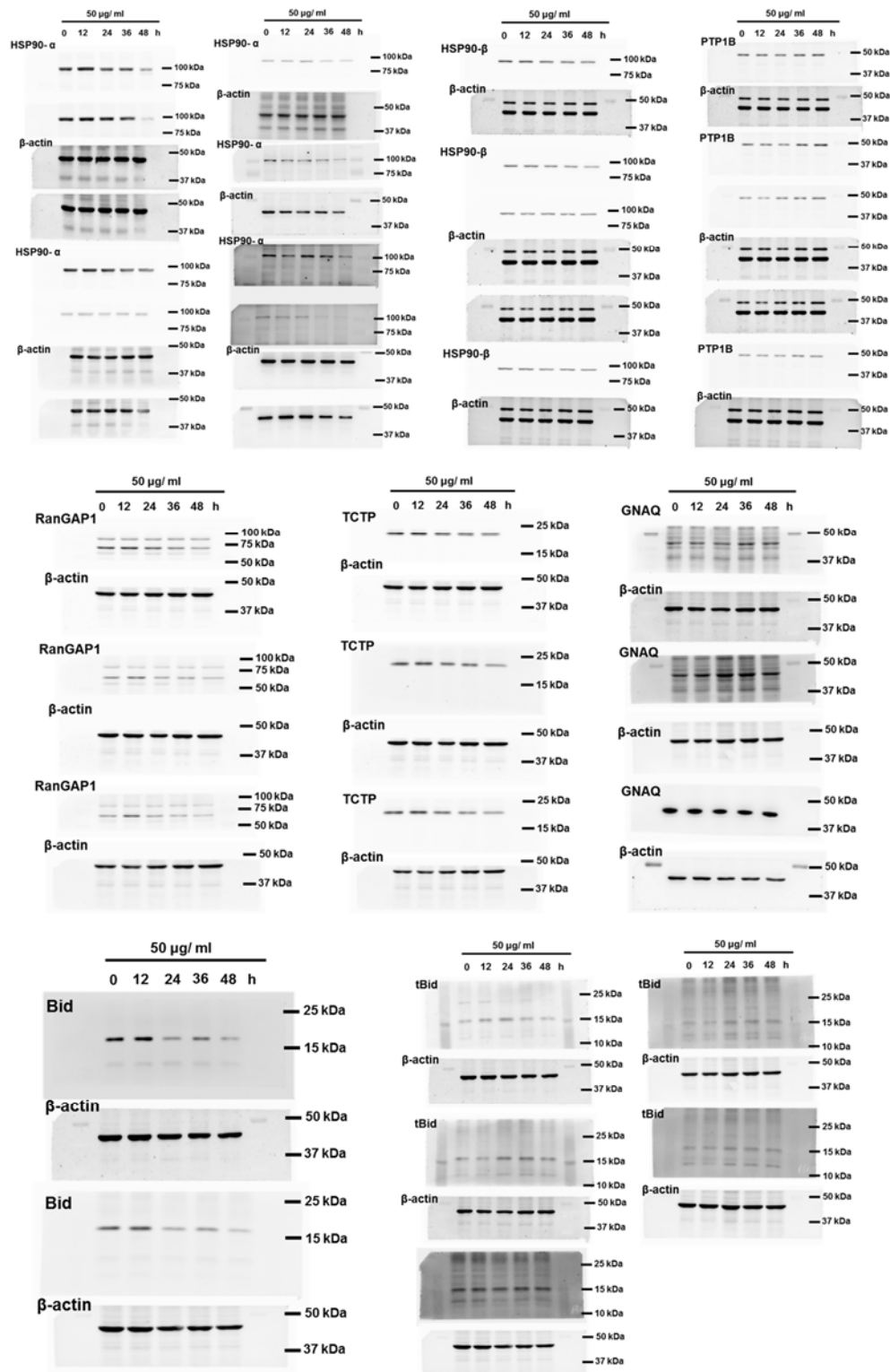

Figure 2b

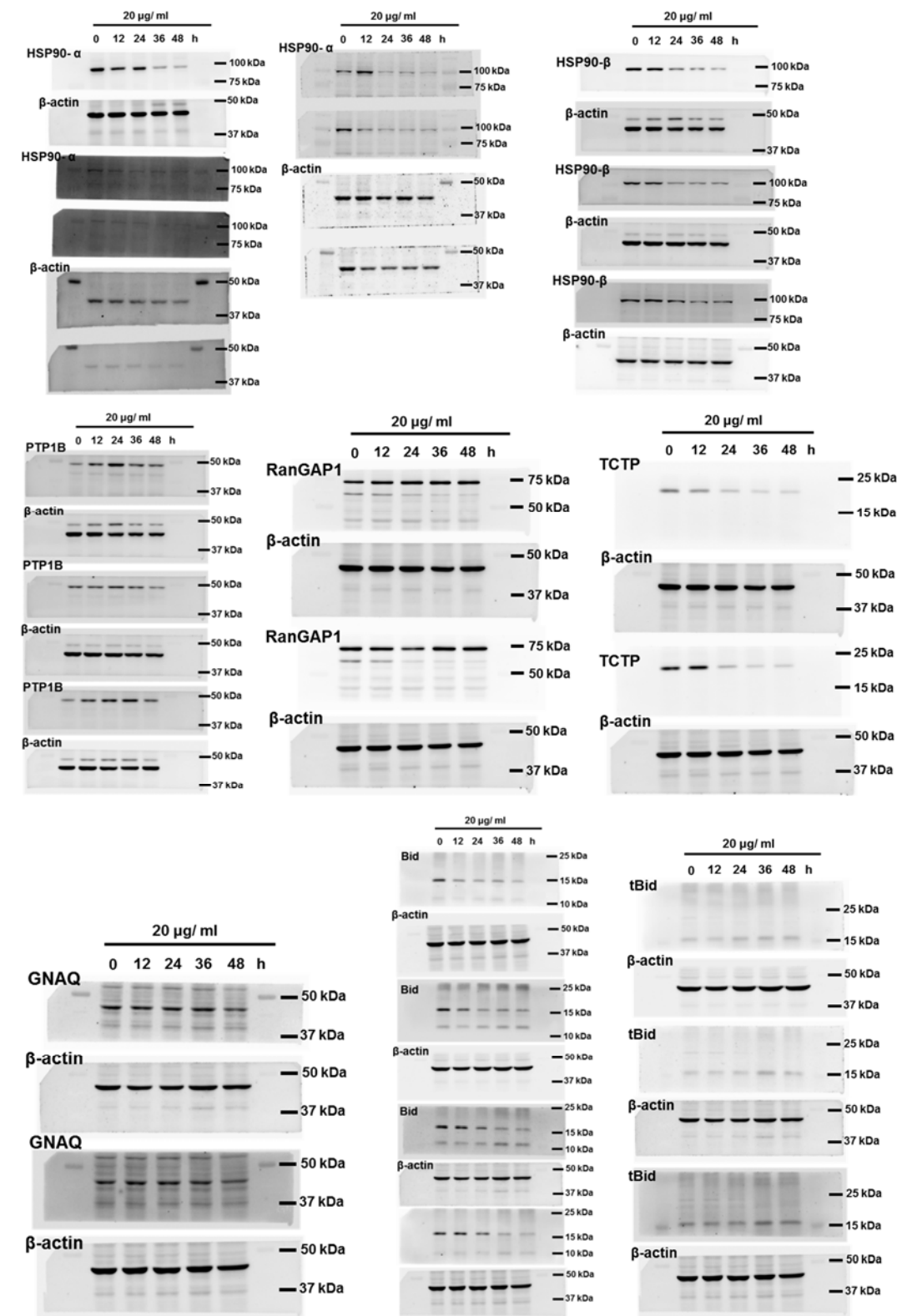

Figure 4a

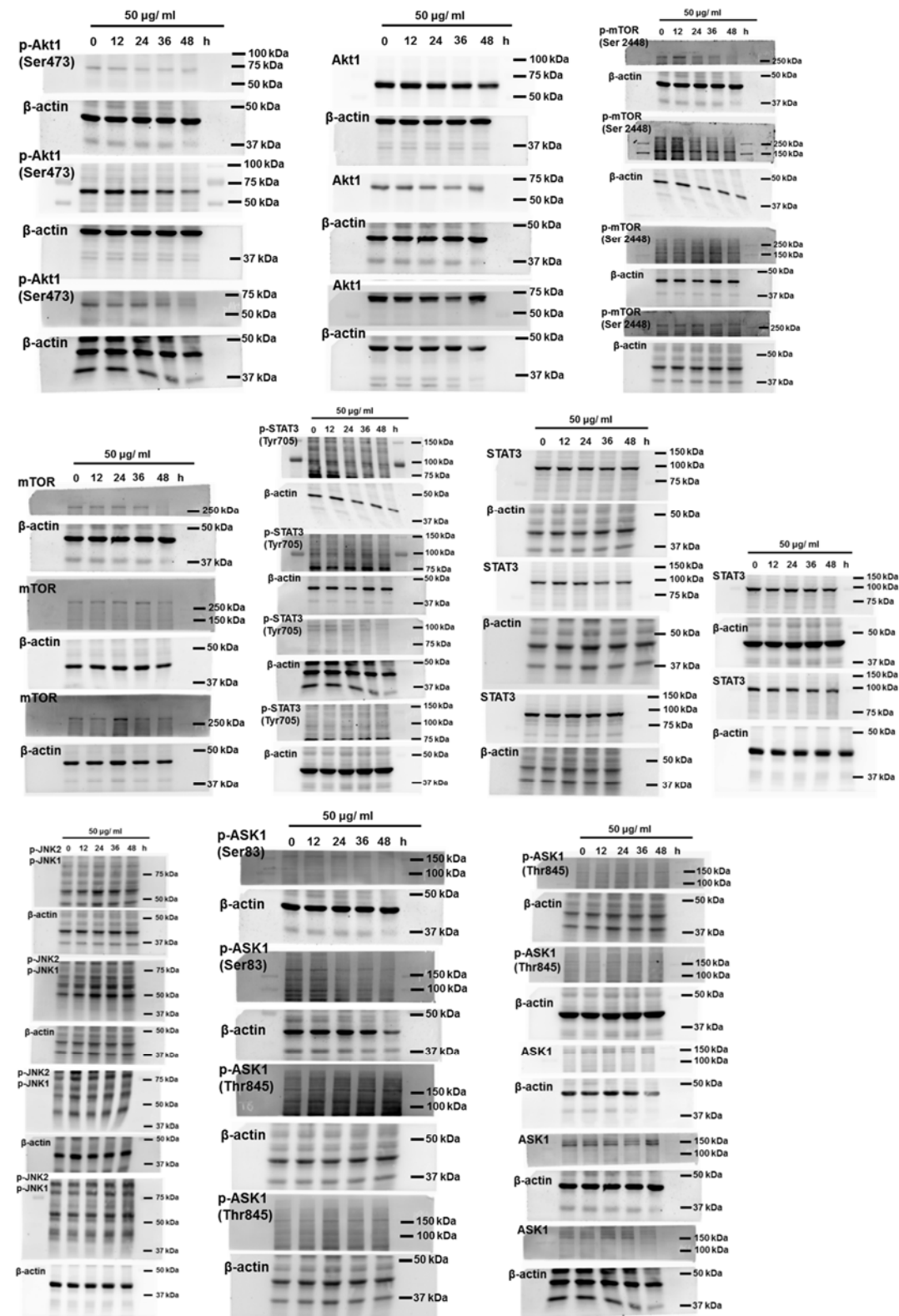

Figure 4b

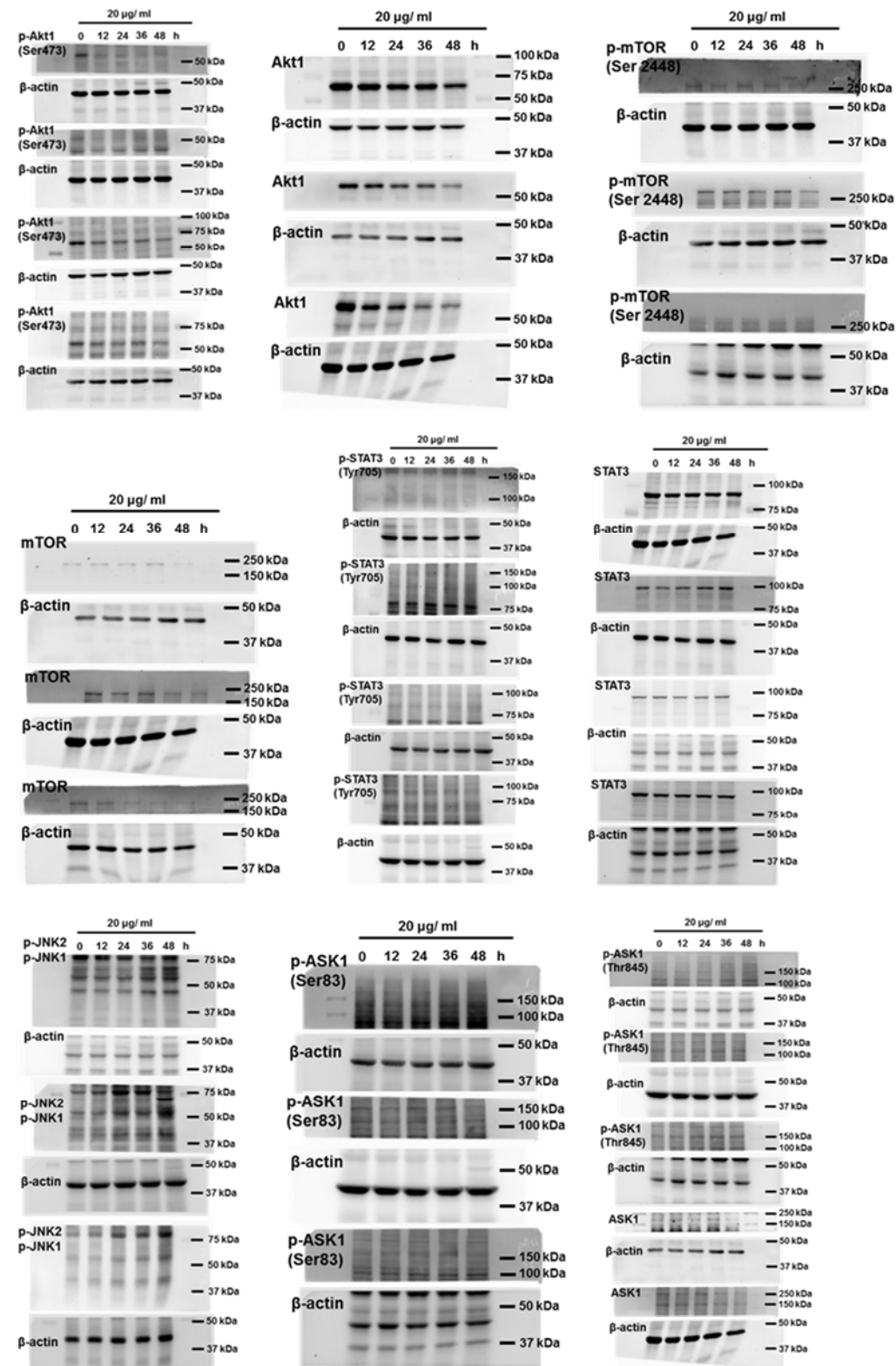

Supplement: Supplementary file 1 [file life-12-01839-s001.zip › Supplementary Materials/Figure S2. Original Blots.pdf]
